# Cytotoxic and Pathogenic Properties of *Klebsiella oxytoca* Isolated from Laboratory Animals

**DOI:** 10.1371/journal.pone.0100542

**Published:** 2014-07-24

**Authors:** Alison Darby, Kvin Lertpiriyapong, Ujjal Sarkar, Uthpala Seneviratne, Danny S. Park, Eric R. Gamazon, Chara Batchelder, Cheryl Cheung, Ellen M. Buckley, Nancy S. Taylor, Zeli Shen, Steven R. Tannenbaum, John S. Wishnok, James G. Fox

**Affiliations:** 1 Division of Comparative Medicine, Massachusetts Institute of Technology, Cambridge, Massachusetts, United States of America; 2 Pharmaceutical Sciences and Pharmacogenomics Graduate Program, University of California, San Francisco, California, United States of America; 3 Section of Genetic Medicine, Department of Medicine, The University of Chicago, Chicago, Illinois, United States of America; 4 Department of Biological Engineering, Massachusetts Institute of Technology, Cambridge. Massachusetts, United States of America; 5 Department of Chemistry, Massachusetts Institute of Technology, Cambridge, Massachusetts, United States of America; Charité-University Medicine Berlin, Germany

## Abstract

*Klebsiella oxytoca* is an opportunistic pathogen implicated in various clinical diseases in animals and humans. Studies suggest that in humans *K. oxytoca* exerts its pathogenicity in part through a cytotoxin. However, cytotoxin production in animal isolates of *K. oxytoca* and its pathogenic properties have not been characterized. Furthermore, neither the identity of the toxin nor a complete repertoire of genes involved in *K. oxytoca* pathogenesis have been fully elucidated. Here, we showed that several animal isolates of *K. oxytoca*, including the clinical isolates, produced secreted products in bacterial culture supernatant that display cytotoxicity on HEp-2 and HeLa cells, indicating the ability to produce cytotoxin. Cytotoxin production appears to be regulated by the environment, and soy based product was found to have a strong toxin induction property. The toxin was identified, by liquid chromatography-mass spectrometry and NMR spectroscopy, as low molecular weight heat labile benzodiazepine, tilivalline, previously shown to cause cytotoxicity in several cell lines, including mouse L1210 leukemic cells. Genome sequencing and analyses of a cytotoxin positive *K. oxytoca* strain isolated from an abscess of a mouse, identified genes previously shown to promote pathogenesis in other enteric bacterial pathogens including ecotin, several genes encoding for type IV and type VI secretion systems, and proteins that show sequence similarity to known bacterial toxins including cholera toxin. To our knowledge, these results demonstrate for the first time, that animal isolates of *K. oxytoca*, produces a cytotoxin, and that cytotoxin production is under strict environmental regulation. We also confirmed tilivalline as the cytotoxin present in animal *K. oxytoca* strains. These findings, along with the discovery of a repertoire of genes with virulence potential, provide important insights into the pathogenesis of *K. oxytoca*. As a novel diagnostic tool, tilivalline may serve as a biomarker for *K oxytoca*-induced cytotoxicity in humans and animals through detection in various samples from food to diseased samples using LC-MS/MS. Induction of *K. oxytoca* cytotoxin by consumption of soy may be in part involved in the pathogenesis of gastrointestinal disease.

## Introduction

*Klebsiella oxytoca* is a non-motile, gram-negative rod-shaped bacterium belonging to the family *Enterobacteriaceae*. *K. oxytoca* is ubiquitous in the environment [1] and can be cultured from the skin, mucous membranes, oropharynx and intestines of healthy humans and animals, as well as a variety of tissues from clinically affected humans and animals [2].

In humans, *K. oxytoca* can be cultured from the stool of 8–10% of healthy adults [3]. Although most *K. oxytoca*-infected individuals remain asymptomatic, *K. oxytoca* is considered an opportunistic pathogen and is now recognized as a clinically significant pathogen associated with nosocomial infections in hospitalized patients, including children and neonates [3], [4], [5]. *K. oxytoca* also is purported to be an etiological agent of antibiotic-associated hemorrhagic colitis (AAHC) in adults and adolescents. Stool cultures of individuals with AAHC contain *K. oxytoca,* but do not contain organisms associated with diarrhea, such as *Clostridium difficle*, *Campylobacter* spp., *Salmonella* spp., *Yersinia* spp., *Shigella* spp., and *E. coli* O157 [6]. AAHC patients develop clinical signs following antibiotic and/or anti-inflammatory therapy; these typically include bloody diarrhea, severe abdominal cramping, and segmental hemorrhagic colitis as visualized by colonoscopy, most commonly in the ascending colon and the cecum [4], [7], [8]. Clinical signs are self-limiting and resolve several days after antibiotics are discontinued. *K. oxytoca* has been cultured from human patients with septicemia, bacteremia, septic arthritis, soft tissue infections, cholecystitis, urinary tract infections, and more recently from colicky neonates [1], [9], [10], [11], [12], [13], [14], [15]. Intestinal overgrowth of *K. oxytoca* has also been observed among children with celiac disease [16].

In animals, *K. oxytoca* has been isolated from apparently healthy sentinel rodents being monitored for pathogens in health surveillance programs and from utero-ovarian infections including suppurative endometritis, salpingitis, perioophoritis and peritonitis in aged B6C3F1 mice on a long term carcinogenicity study [17], [18]. *K. oxytoca* was also cultured from cases of suppurative otitis media, urogenital tract infections and pneumonia in C3H/HeJ and NMRI-Foxn1 (*nu*) mice, LWE.1AR1 rats, and in mole voles [19]. Additionally, *K. oxytoca* was recently cultured from three breeding colonies of NOD.Cg-*Prkdc^scid^ Il2rg^tm1Wjl^*/SzJ (NSG) mice with chronic renal inflammation and ascending urinary tract infections [20]. An outbreak of *K. oxytoca* enterocolitis on a rabbit farm was also reported [21]. In 2006, Hogenauer *et al.* developed a model of AAHC by administering amoxicillin-clavulanate followed by orally infecting rats with a strain of *K. oxytoca* cultured from a patient with AAHC. The Sprague-Dawley rats developed intestinal lesions primarily in the cecum. *K. oxytoca* was subsequently cultured from the rats with enterocolitis, fulfilling Koch’s postulates and establishing the positive association between AAHC and *K. oxytoca* infection [7]. The ability of the human strain of *K. oxytoca* to colonize and cause disease in rats experimentally [7] also emphasizes the cross-species infecting capability of *K. oxytoca*. Although the experimental infection study suggests that human strains of *K. oxytoca* can infect rats, no evidence to date suggests that *K. oxytoca* strains from rats or from other animal species can naturally infect humans and vice versa.

*K. oxytoca* strains associated with colitis and mucocutaneous infections in humans can produce a cytotoxin, which may, in part, explain *K. oxytoca* pathogenesis [7]. The cytotoxin described in 1989 and 1992 caused cell rounding and cell death when applied *in vitro* to various cell lines including HEp-2, HeLa, CHO, and Vero cells [22], [23]. However, cytotoxin production in *K. oxytoca* isolated from animals has not been described [18], [19], [20]. Furthermore, despite the known clinical effects of *K. oxytoca* in humans and animals and the discovery of cytotoxin production in this bacterium, a complete portrait of its pathogenic mechanisms is lacking. We hypothesized that - similar to *K. oxytoca* strains in humans - *K. oxytoca* isolates of animal origin also produce cytotoxin and that *K. oxytoca* contain a repertoire of genes that promote pathogenicity. To test this hypothesis, we obtained and characterized antibiotic resistance patterns in *K. oxytoca* strains from various animal species and investigated their ability to produce cytotoxin under various growth conditions using standard *in vitro* cytotoxicity assays previously described [22], [23]. Furthermore, we sequenced the genome of a cytotoxin-positive, clinical isolate from diseased tissue of a mouse and performed *in silico* analysis to gain insight into its potential pathogenic properties.

**Figure 1:**
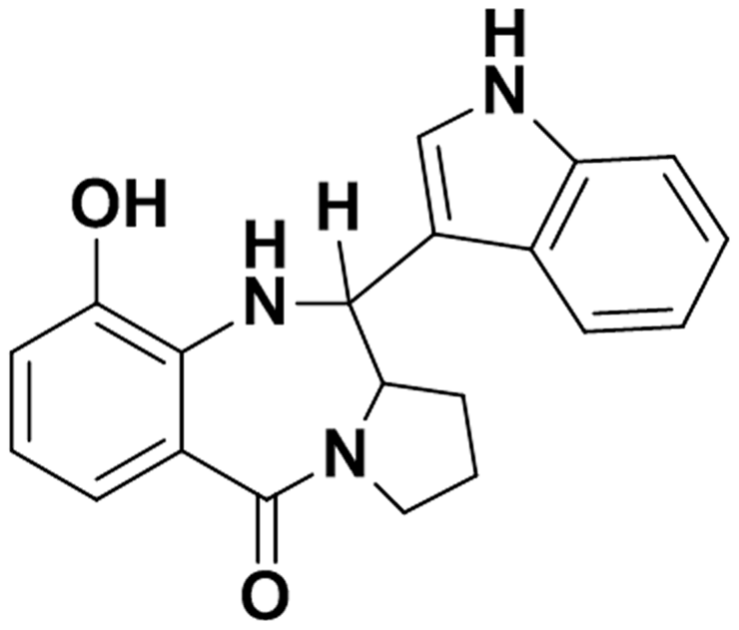
Tilivalline structure, chemical formula, and mass.

Some of the natural exotoxins produced by *Streptomyces* and *Micrococci* species have a common pyrrolo-benzo-diazepine (PBD) moiety [24], [25]. These area typically cytotoxic class of compounds consisting of mainly three rings [24]. Tilivalline (Figure 1), for example, is cytotoxic to mouse leukemia L1210 cells [25], [26]. However, the molecular mechanisms of cytotoxicity induced by these compounds are a matter of active investigation. With the previous results in mind, we suspected that the cytotoxic activity present in the supernatant of laboratory animal strains of *K. oxytoca* might also be tilivalline. We therefore set out to determine if tilivalline was present in the culture broth of cytotoxic-positive strains of *K. oxytoca*, and correspondingly absent in negative strains under identical growth conditions, and ascertain whether tilivalline induced cell abnormalities and death in a cytotoxicity assay.

## Materials and Methods

### Strains of *K. oxytoca*

Sixty four isolates of *K. oxytoca* isolated from various laboratory animal species were acquired from several sources and used in this study. Of the 64 isolates, 48 were from mice (obtained from Charles River Laboratories (CRL), Jackson Laboratory, MIT Comparative Medicine diagnostic lab, and National Institute of Environmental Sciences); 4 isolates were from rats (CRL), 8 from non-human primates (CRL), 3 from pigs (CRL), and 1 from a guinea pig (CRL). In addition, a non-toxigenic *K. oxytoca* strain, ATCC 13182 [27], was obtained from American Type Culture Collection (ATCC) and used as a control.

### Verification of *K. oxytoca* isolates

The identities of all 64 strains, previously identified by outside sources as *K. oxytoca*, were verified by a combination of API 20E test (bioMerieux, Marcy l'Etoile, France), polygalacturonase (*pehX*) gene amplification, and 16S rRNA gene sequencing. *K. oxytoca* DNA was used for subsequent molecular analyses with the Roche High Pure PCR Template Kit according to the manufacturer’s recommendations (Roche Applied Science, Indianapolis, IN). For *pehX* gene amplification, forward primer PEH C (5’ GAT ACG GAG TAT GCC TTT ACG GTG -3’) and reverse primer PEH D (5’- TAG CCT TTA TCA AGC GGA TAC TTG -3’) (Integrated DNA Technologies, San Diego, CA) [28] were used. PCR amplification was performed using PuReTaq Ready-To-Go PCR Beads (GE Healthcare, Piscataway, NJ) as previously described [28]. The PCR products were resolved on 2% agarose gel by electrophoresis and stained with ethidium bromide before visualization. Primer 9F (positions 9 to 27 in the forward direction) and primer 1541 (positions 1525 to 1541 in the reverse direction) were used to amplify the 16S rRNA genes from *K. oxytoca* as previously described [29]. The PCR products were then purified using the QIAquick PCR purification kit (Qiagen, Valencia, CA) and sequenced with an ABI Prism cycle sequencing kit (BigDye Terminator cycle sequencing kit) on an ABI 3500 genetic analyzer (Applied Biosystems, Foster City, CA). Sequences were compared directly with the National Center for Biotechnology Information (NCBI) Genbank nucleotide database by BLAST search.

### Antimicrobial Susceptibility

Antimicrobial susceptibility testing was performed using the disk diffusion method according to the standards set by Clinical and Laboratory Standards Institute. Briefly, tryptic soy broth (TSB) (BD, Franklin Lakes, NJ) was inoculated with *K. oxytoca* from 24-hour blood agar cultures and incubated at 37°C for 1 hr. The cultures were spread evenly on Meuller Hinton agar and antibiotic discs were aseptically placed on the agar surface. The following antibiotic discs were used: ampicillin 10µg, cephalothin 30µg, amoxicillin/clavulanic acid 20/10µg, trimethoprim/sulfamethoxazole 1.25/23.75µg, enrofloxacin 5µg, and gentamicin 10µg (Remel, Lexington, KS). Samples were incubated aerobically for 18–24 hours at 37°C and the zones of inhibition were measured to the nearest millimeter. Measurements were categorized as susceptible, intermediate susceptibility, or resistant based on the established values for *Klebsiella* spp. reported by the manufacturer (BBL, Becton, Dickinson and Company, Sparks, MD).

### Bacterial Cultures

To propagate *K. oxytoca*, frozen stocks of bacteria were seeded on blood agar (Remel) and grown for 24 hours at 37°C. To investigate the effects of environmental conditions on cytotoxin production, a single colony of *K. oxytoca* from a 24-hour culture was inoculated into 8 ml of tryptic soy broth (TSB) and then subjected to one of four conditions of variable oxygen content. The first condition was a previously established growth condition used in investigating cytotoxin production in human isolates of *K. oxytoca* [22], [23]. This involved culturing *K. oxytoca* in a 37°C incubator without agitation for 16 hours with 5% CO_2_. The second method was similar to the first method except CO2 was not provided and gentle agitation at 100 rpm was implemented The third condition was microaerobic condition (10% CO_2_, 10% H_2_, 80% N_2_) in GasPak (BD, Franklin Lakes, NJ) jars, as previously described [30]; the culture was incubated at 37°C with gentle agitation at 100 rpm for 16 hours. The fourth method was cultivation under anaerobic conditions at 37°C for 16 hours with agitation using AnaeroPack anaero (Mitsubishi Gas Chemical Co., Inc., Chiyoda, TKY). To determine the effect of various growth media on cytotoxin production, a single colony of *K.oxytoca* was inoculated into 8 ml of various types of growth media (see **Media Preparation**) and incubated for 16 hours at 37°C under microaerobic condition with gentle agitation. The incubation duration of 16 hours was chosen based on the report that cytotoxin production reaches maximum levels at the end of the exponential growth phase, approximately 16 hours after inoculation into TSB liquid media [27]. At this stage, the liquid cultures had OD _660 nm_ measurement range of 1.4–1.6.

### Cytotoxicin Assays

HEp-2 (ATCC-CCL-23) and/or HeLa S3 (ATCC-CCL-2.2) cell lines were used to investigate cytotoxic activity in *K.oxytoca* as previously described [22], [23]. Initially, both HEp-2 and HeLa cells were used to determine cytotoxity of all *K. oxytoca* strains. Only HEp-2 cells were used in all other subsequent experiments. Cell lines were maintained as recommended by ATCC with Hyclone MEM/EBSS + 2.0 mM L-glutamine, + Earle’s balanced salts (HyClone Laboratories Inc., Logan, Utah) and 10% fetal bovine serum (Atlas Biologicals, Fort Collins, CO). Approximately 1.0×10^4^ cells suspended in 500 µl of cell culture media were seeded into 24-well cell culture plates and incubated for 1–2 hours in 37°C with 5% CO_2_. To investigate cytotoxin production among different *K. oxytoca* strains, 100 µL of supernatant obtained from liquid culture (see **Bacterial Cultures**) were added to the wells containing HEp-2 or HeLa cells. To obtain the supernatant, the bacteria liquid culture was centrifuged at 8,000×g for 5 minutes. The resulting supernatant was then filtered through 0.2 µm Acrodisc syringe filters (PALL Corporation, Port Washington, NY) to exclude bacteria. Supernatants were either used immediately or stored at −20°C until assayed. After 48 hours of incubation in 37°C incubator aerated with 5% CO_2_, the percentage of cells adhering to the bottom of the well of 24-well plates (% confluency) indicative of live cells was assessed using an Olympus CK2 inverted phase contrast microscope at 4, 10 and 30× magnification. To facilitate visualization of the cells and to determine the total area of cells adhered to the well, Diff Quick straining was also performed. Cytotoxin-positive samples were characterized as <50% confluency i.e., greater than 50% cell rounding and detachment, as compared to negative control samples (TSB media only or supernatant of a non-toxigenic *K.oxytca* strain, ATCC 13182) [27]. Negative control samples had a monolayer of cells with minimal cell rounding or detachment and confluency in the range of 80% to nearly 100%. The cytotoxicity assay was performed at least three times in duplicate wells for each *K. oxytoca* to confirm cytotoxin production status.

### Heat Inactivation and proteases, trypsin, DNase, and RNase treatment of Cytotoxin

A 24-hour culture of *K. oxytoca,* 09-7231, was inoculated into TSB liquid media and grown under microaerobic conditions with gentle agitation for 16 hours. One milliliter of filtered supernatant was obtained and subjected to heat treatment by boiling in a test tube for 20 minutes or treated with protease (1 mg/ml of supernatant) or trypsin (0.5 units/100 µl) at 37 °C with continuous shaking (70 rpm) for 2 hours. For protease and trypsin treatments, the treated supernatant was subjected to centrifugation at 3000 g for 1 minute to remove protease and trypsin agarose. Protease and trypsin agarose (Sigma, St Louis, MO) was reconstituted according to manufacturer’s recommendations. DNase (20 units/ml, Sigma) and RNase (10 µg/ml, Sigma) treatments of the supernatant were performed by incubating at room temperature for 2 hours [31]. One hundred μl of treated supernatant was used for the cytotoxicity assays [32]. As a control, heated TSB broth and protease-, trypsin-, DNase-, and RNAse-treated TSB broth were used. All assays were performed in 3 independent experiments.

### Media preparation

Three different types of media: soy extract only, casein extract only, and TSB, were prepared. Media containing soy extract only consists of peptic digest of soybean meal (Bacto Soytone, BD; 20 g/L of water), dextrose (2.5 g/L), and NaCl (5 g/L). Media containing only casein extract consists of pancreatic digest of Casein (Bacto Tryptone; 20 g/L of water), dextrose (2.5 g/L) and NaCl (5 g/L). TSB contains pancreatic digest of casein (Bacto Tryptone 17 g/L), Bacto Soytone (3 g/L), dextrose (2.5 g/L), and NaCl (5 g/L).

### Molecular weight determination

Supernatant from the mouse clinical *K.oxytoca* isolate, 09-7231-1, grown under microaerobic conditions as described above, was subjected to filtration using Centricon Ultracel YM 3 kD and 30 kD membranes (Millipore Corporation, Billerica, MA). Fractions obtained were then assessed in the *in vitro* cytotoxicity assay to determine biological activity.

### Global gene expression analysis by microarray

Human GE 4×44K V2 Microarray (Ilumina) was used to investigate global gene expression changes associated with the *K. oxytoca* toxin on HEp-2 cells. 1×10^6^ HEp-2 cells suspended in 1 mL culture medium were added in each well of the 6-well tissue culture plate. Two hundred microliters of supernatant from the cytotoxin positive *K. oxytoca*, 09-7231, which was subjected to 3K protein separation membrane, was added to each well. As a control, HEp-2 cells were treated with TSB media subjected to 3K protein separation membrane. Six hour post treatment, the cells were collected and RNA extracted using a combination of Trizol and RNA extraction kit (Qiagen). Samples from 4 independent toxin treated and control tissue culture wells were used for microarray analysis. RNA samples were processed by MIT Biomicro Center (http://openwetware.org/wiki/BioMicroCenter). The samples were labeled according to Agilent Two-Color Microarray-based Gene Expression Analysis Protocol (Low Input Quick Amp Labeling Kit) (http://www.chem.agilent.com/library/usermanuals/public/G4140-90050_GeneExpression_TwoColor_6.6.pdf). RNA was hybridized to Human GE 4×44 v2 microarray (Agilent Technologies, Santa Clara, CA). Arrays were washed and scanned using the G2565 Microarray Scanner (Agilent Technologies). For visualization, mRNA expression analysis and other bioinformatics analyses of the microarray data, we utilized Agilent’s GeneSpring GX software suite. We performed differential expression analysis using the t-test against the null hypothesis of no change on each of ∼ 34000 probes. Multiple testing correction for the differential expression analysis was done using the Benjamini-Hochberg method. We conducted Gene Ontology functional annotation enrichment analysis using DAVID as well as pathway enrichment analysis in GeneSpring GX for the genes that showed significant differential expression (p_adjusted_<0.05). In these enrichment analyses, p_adjusted_<0.05 (using the Benjamini-Hochberg procedure) was defined as significant.

### Tilivalline detection by LC-MS and LC-MS/MS

HPLC grade chloroform and acetonitrile, formic acid for LC-MS, and LC-MS/MS were purchased from Sigma Aldrich. The supernatant from culture broth was extracted with chloroform, dried, and resuspended in 50 µL 2% acetonitrile containing 0.1% formic acid. Samples were analyzed with an Agilent 1290 ultra-high pressure liquid chromatography system (Waldbronn, Germany) interfaced with a 6530 quadrupole time-of-flight (QTOF) mass spectrometer with a Jetstream electrospray ionization source and MassHunter workstation (version B.06). The column was an Agilent C18 (2.1×50 mm, 1.8 µm). Mass spectra were acquired in the positive ion mode from *m/z* 60 to *m/z* 1000 at 4 scans per second. The ion spray voltage was 3,800 V, and the heated capillary temperature 350°C. Two reference masses (*m/z* 121.0509 (C_5_H_4_N_4_) and *m/z* 922.0098 (C_18_H_18_O_6_N_3_P_3_F_24_) were infused during the runs. The column thermostat and autosampler temperatures were 50°C and 6°C, respectively. The solvents were water with 0.1% formic acid and acetonitrile containing 0.1% formic acid. The gradient was 2% to 90% B over 5 minutes, at 0.5 mL/min.

MS/MS was generated on an Agilent QTOF 6530 mass spectrometer (Santa Clara, CA) to further confirm the identity of the metabolites. The column and gradients were the same as those used for metabolite profiling. The AJS-ESI source was set as MS scan mode. A targeted list, which included previously determined exact masses according to results obtained with extracted ion chromatogram (EIC), was generated for fragmentation.

### Isolation of tilivalline

The supernatant from the growth media (1 L) was extracted in 500 mL portions, each with 250 mL of chloroform (3X). The combined organic phase was dried (anhydrous sodium sulfate), concentrated under reduced pressure, and the crude product was semi-purified by automated reverse phase flash chromatography (Biotage) using a water/methanol gradient. The fractions eluting at 65% methanol were collected, concentrated under reduced pressure and re-dissolved in 5% acetonitrile in water. Aliquots were further purified by preparative HPLC with an Agilent Technologies model 1100 HPLC system equipped with a photodiode array UV detector (Wilmington, DE). UV absorbance was monitored at 254 nm. A semi-preparative Phenomenex Luna C18 (25 cm×9.4 mm, 10 µm) column was eluted with a linear gradient of 0.1% formic acid in water (A) and 0.1% formic acid in acetonitrile (B) at a flow rate of 2.5 mL/min. Solvent composition was initially at 10% for 5 min, changed to 50% over 10 min, changed from 50% to 70% B over 15 min, and then further to 95% B over 2 min, held for 8 min, followed by returning to 10% B over 2 min for a total run time of 42 min. The column was equilibrated for 10 minutes between injections. The fractions eluting around 21.4 minutes (Figure S5) were collected and combined for toxicity evaluation and NMR analysis (Figure S4).

### Genome sequencing and *In Silico* Analysis

To further explore the pathogenic mechanisms of *K. oxytoca*, the genome of the mouse *K. oxytoca* strain, 09-7231-1, was fully sequenced in collaboration with the Broad Institute of Harvard and MIT (*Klebsiella* group Sequencing Project (http://www.broadinstitute.org/)). The full genomic sequence was deposited at the National Center for Biotechnology Information (NCBI accession: PRJNA52135). Using a comprehensive set of reference “heat-labile toxins” obtained from NCBI (n = 920), we conducted homology searches and used the BLOSUM80 scoring matrix [33], which was created based on local alignments of highly homologous (≥ 80%) proteins. We assumed that domains contributing to the virulence and heat liability should be highly conserved between bacterial strains since these key functions are essential to their fitness. We varied the E-value threshold to the maximum value of 10, as it is possible to have a high percentage of homologues in BLAST results with E-values ranging from 0 to 10. Indeed, a study by Boekhorst. *et al*., revealed that, when querying annotated protein families in the PFAM database [34], [35], 65% of BLAST hits with E-values greater than 1e-03 and less than 10 were known homologues of the query protein according to PFAM. Additionally, the same study found that 43% of BLAST hits with E-values greater than 1 and less than 10 were known homologues [35]. Using the thresholding approach to prioritize hits, we aimed to include all genes that could be potential *K. oxytoca* cytotoxin proteins. We performed in-depth analyses of the subset of toxins that met an E-value cutoff of 1. In this last set of genes, we included the toxin, PaxA, because one of its transporters, PaxB, had produced statistically significant results in our first round of BLAST analyses. We calculated the molecular weights of the subset of toxins showing homology at the chosen E-value threshold, using the “Compute pl/Mw” tool on the ExPASy server [36].

### Statistical analysis

We tested whether there was a significant relationship between the percentages of *K. oxytoca* strains producing cytotoxin in one growth condition version another growth condition using a Chi-squared test with 2 degrees of freedom.

## Results

### Animal isolates of *K. oxytoca*

Sixty four isolates from various animals were confirmed as *K. oxytoca* (Table 1). Of the 48 mouse isolates, 14/48 (29%) were cultured from the respiratory tract, 19/48 (40%) from feces of asymptomatic mice, 4/48 (8%) isolates from abscesses in the lung, palpebral conjunctiva, and a tumor, and 11/48 (23%) from unknown tissues or sites. Of the 4 rat isolates, 1 was isolated from mammary lesions, while the rest were isolated from unknown tissue. Three of 8 isolates of the non-human primates were obtained from feces; tissue origins of the 5 remaining primate isolates, the pig and guinea pig isolates were unknown (Table 1).

**Table 1:** Sources and origin of *Klebsiella oxytoca* used in this study.

Species	N =	Source	Tissue/Media
G. Pig	1	Diagnostic Lab	Unknown
Mouse	19	Diagnostic Lab 1	Feces
Mouse	1	Diagnostic Lab 1	Lung Abscess
Mouse	12	Diagnostic Lab 1	Nasal Culture/Flush
Mouse	1	Diagnostic Lab 1	Palpebral Abscess
Mouse	6	Diagnostic Lab 1	Unknown
Mouse	2	Commercial Vendor	Unknown
Mouse	2	Diagnostic Lab 2	Abscess, nu/nu mouse
Mouse	3	Diagnostic Lab 2	Unknown
Mouse	2	Diagnostic Lab 3	Nasal Flush
Rat	1	Diagnostic Lab 1	Mammary Lesion
Rat	3	Diagnostic Lab 1	Unknown
Simian	3	Diagnostic Lab 1	Feces
Simian	5	Diagnostic Lab 1	Unknown
Swine	3	Diagnostic Lab 1	Unknown
Ref.	1	ATCC 13182	Human Pharyngeal tonsil
Total	65		

Given that *K. oxytoca* isolates have varying pathogenic potentials which could, in part, be attributed to their antibiotic resistant signatures (Table 2), we determined the antibiotic resistant characteristics of each of these isolates. Overall, 27/64 (42%) of the isolates were resistant to cephalothin, 5/64 (8%) to amoxicillin clavulanate, 4/64 (6%) to trimethoprim/sulfamethoxaxole, 4/64 (6%) to enrofloxacin, and 2/64 (3%) to gentamicin. All 4 isolates that were resistant to trimethoprin/sulfamethoxole, enrofloxacin, and 2 isolates that were resistant to gentamicin were exclusively from non-human primates. All *K. oxytoca* strains were resistant to ampicillin, a key characteristic of this β-lactamase organism [27] (Table 2).

**Table 2:** Antimicrobial resistance of *K. oxytoca.*

Species of origin	(# of samples)	Ampicillin	Cephalothin	Amoxicillin/Clavulanate	Trimethoprim/Sulfamethoxaxole	Enrofloxacin	Gentamicin
G. pig (1)		1	0	0	0	0	0
Mouse (48)		48	23	4	0	0	0
Rat (4)		4	1	1	0	0	0
Simian (8)		8	0	0	4	4	2
Swine (3)		3	3	0	0	0	0

Antibiotic discs: ampicillin10µg; cephalothin 30µg; amoxicillin/clavulanic acid 20/10µg; trimethoprim/sulfamethoxazole 1.25/23.75µg; enrofloxacin 5µg; and gentamicin 10µg.

### Cytotoxin production of *K*. *oxytoca* isolates of animals

**Figure 2:**
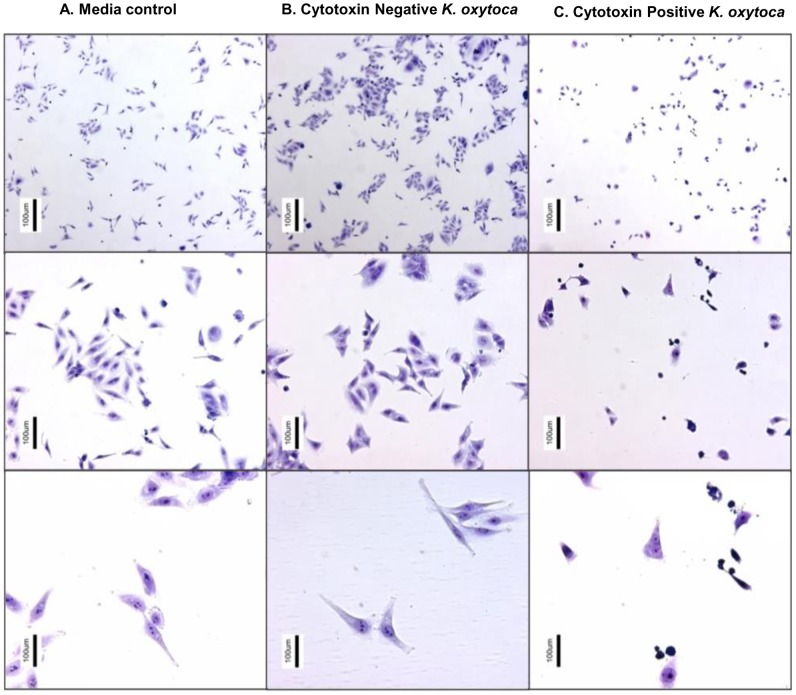
Animal isolates of *K. oxytoca* produce cytotoxin. HEp-2 cell culture inoculated with *K. oxytoca* supernatant and media control at 4, 10, and 30× magnifications. **A)** Media control showing normal morphology of cells stained with Diff-quick after 48 hours of incubation. **B)** *K. oxytoca*, ATCC 13182, isolate negative for cytotoxin production showing normal cell morphology. **C)** *K. oxytoca* isolate, 09-7231-1, positive for cytotoxin production showing abnormal cell morphology, decreased concentration of attached cells, small round cells, and multinucleated cells.

To determine whether *K. oxytoca* strains isolated from animals produced cytotoxin, we performed *in vitro* cytotoxicity assays using both HEp-2 and HeLa S3 cells (See **Materials and Methods**). All *K. oxytoca* strains were grown using the previously reported culturing methods [22], [23], which were also described in **Material and Methods**. Of the 64 isolates of *K. oxytoca* investigated, 18 (28%) isolates were found to induce greater than 50% cell abnormality and death (< 50% confluency), fulfilling our criteria for classifying the bacteria as cytotoxin positive (Table 3). As expected, the non-toxigenic strain of *K.oxytoca*, ATCC 13182, produced supernatant that did not have any obvious effects on HEp-2 and HeLa cells (Figure 2). Compared to HeLa cells, HEp-2 cells were more susceptible to cytotoxin produced by *K. oxytoca* isolates investigated (data not shown); therefore, HEp-2 cells were used in the subsequent experiments.

**Table 3:** Cytotoxin of *K. oxytoca* varies with different growth conditions.

Species of origin	Number of strains	Supernatant Growth Conditions		
		Aerobic w/5% CO2 (AR)	Aerobic w/agitation (AG)	Microaerobic w/agitation (MG)
		(No. + (%))	(No. + (%))	(No. + (%))
**Mouse**	48	10 (20.8)	20 (41.7)	18 (37.5)
**Rat**	4	3 (75)	3 (75)	3 (75)
**Simian**	8	3 (37.5)	3 (37.5)	3 (37.5)
**Swine**	3	1 (33.3)	1 (33.3)	1 (33.3)
**G. pig**	1	1 (100)	1 (100)	1 (100)
**ATCC 13182**	1	0 (0)	0 (0)	0 (0)
**Total**	**65**	**18 (28)**	**28 (45)**	**26 (44)**

### Growth conditions influence cytotoxin production in *K. oxytoca*

Previous investigations on cytotoxin production in strains of *K. oxytoca* isolated from human patients were performed under static aerobic conditions i.e., in TSB liquid media incubated in 37°C incubator without agitation [4], [22], [23]. Because *K. oxytoca* can thrive in various environmental conditions (e.g. on the skin where O_2_ tension is high relative to the gastrointestinal tract), we hypothesized that cytotoxin production of *K. oxytoca* may be differentially regulated depending on environmental conditions[37], [38], [39], [40]. Therefore, in addition to the static culturing conditions commonly used (AR), we subjected all *K. oxytoca* isolates to three additional *in vitro* conditions – aerobic conditions with gentle agitation without CO_2_ (AG), microaerobic conditions with gentle agitation (MG), and anaerobic conditions (AN) (See **Materials and Methods**) – and evaluated whether these various conditions have different effects on cytotoxin production. Gentle agitation was implemented for aerobic and microaerobic conditions to ensure maximum exposure of bacteria to O_2_ in aerobic conditions as well as CO_2_, H_2_, and N_2_ present in the microaerobic conditions. While 10/48 (∼20%) of mouse isolates were found to be cytotoxin positive under AR, 20/48 (∼42%) and 18/48 (∼38%) were cytotoxin positive under AG and MG, respectively (Table 3). This increase in the number of cytotoxin positive strains was statistically significant between AR and AG (p = 0.03), whereas the difference in AR and MG showed a trend toward significance (p = 0.07). Of the 20 mouse isolates that were cytotoxin positive under AG, only 7 of these isolates were also considered as cytotoxin positive under AR (Table 3). The remaining 3 mouse isolates that were cytotoxin positive under AR were cytotoxin negative under both AG and MG. All 3 rat isolates, a guinea pig isolate, and a pig isolate that were all cytotoxin positive under AR were also cytotoxin positive under both AG and MG (Table 3). In contrast, a single *K. oxytoca* strain isolated from a non-human primate was positive under AR; however, it was negative on both AG and MG. Another *K. oxytoca* strain from a non-human primate was cytotoxin positive under both AG and MG, but was cytotoxin negative under AR (Table 3).

To investigate the effects of AN on cytotoxin production, 34 selected isolates of *K. oxytoca* (mice: 25; non-human primates: 4; guinea pigs: 1; pigs: 1; rats: 3), all of which produced cytotoxin when incubated under AG and MG, were grown under AN. Only 8/34 (23%) (3 mice; 3 rats; 1 guinea pigs; 1 pig) were positive for cytotoxin production (data not shown). Together, these results suggest that AG and MG have a stronger positive effect on cytotoxin production in *K. oxytoca* as compared to AR and AN. Moreover, these results suggest that cytotoxin production among different strains of *K. oxytoca* is under complex environmental regulations.

Intriguingly - although perhaps coincidentally - 13/22 (59%) *K. oxytoca* isolates from feces were cytotoxin positive under at least one of the culturing conditions; virtually the same percentage (9/14; 64%) from the respiratory tract were cytotoxin positive. Of the four clinical isolates of *K. oxytoca* (3 mouse isolates isolated from either a lung abscess, a palpebral abscess, and a tumor abscess, and a single isolate isolated from mammary lesions in a rat (Table 1), only one isolate (mouse isolate, 09-7231-1, from a tumor abscess) was positive for cytotoxin.

### Soy broth induces strong toxin production in *K. oxytoca*

**Figure 3:**
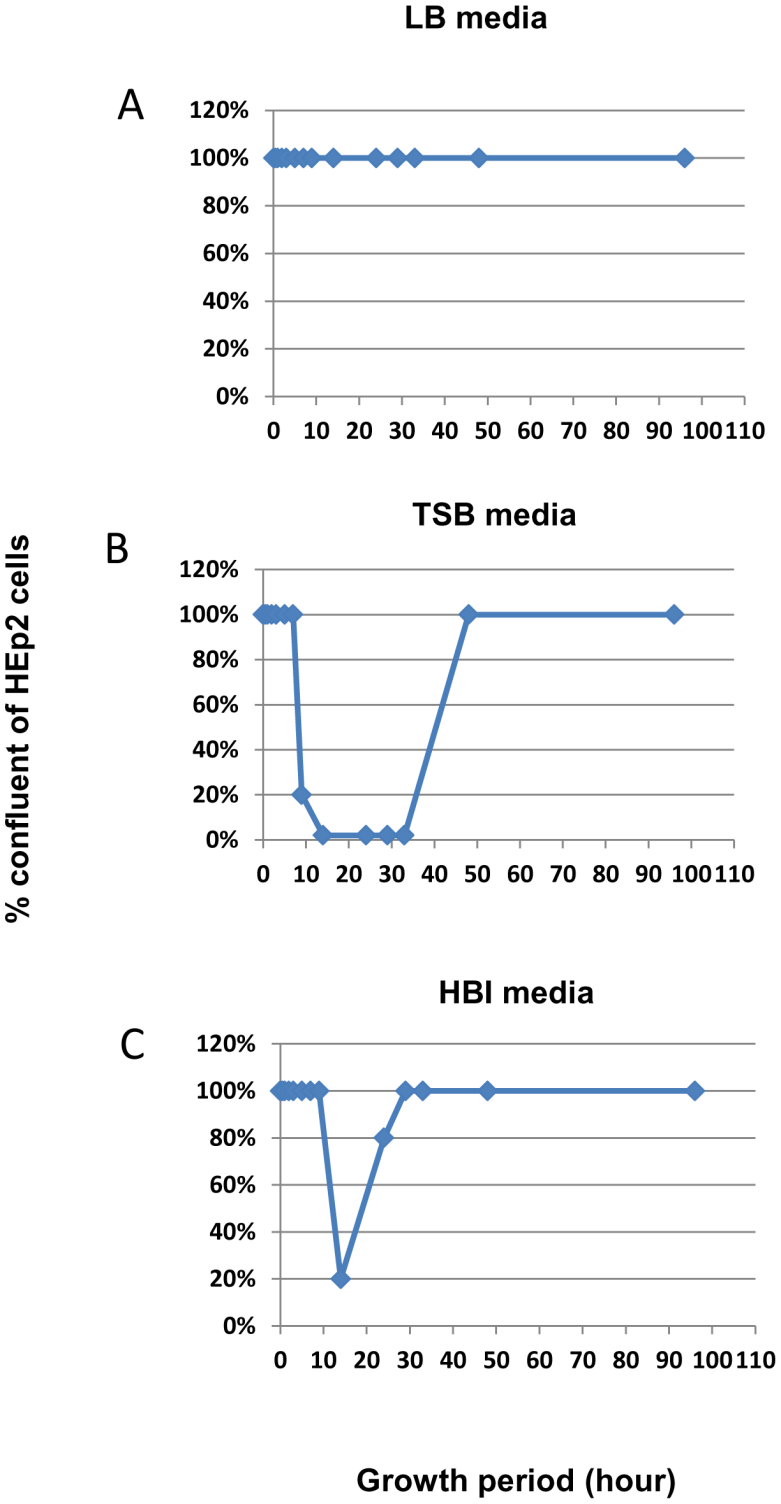
Temporal pattern of cytotoxicity in various conventional bacterial growth media. *K. oxytoca*, 09-7231-1, was cultured in **A)** LB; **B)** TSB; and **C)** HBI liquid media. At various time points, the supernatant was collected and used in the cytotoxicity assay using HEp-2 cells. TSB media has the strongest cytotoxin induction capability compared to LB and HBI media. The degree of cytoxicity is indirectly proportional to the level of monolayer confluency i.e., the higher % the confluency, the lower the cytotoxity and vice versa.

Toxin production in enteric bacteria, such as *Clostridium difficile*, has been shown to be regulated by nutrients [41]. To investigate this possibility in *K. oxytoca*, *K. oxytoca*, 09-7231-1, was grown in various growth media with varying nutrient compositions (See **Materials and Methods**). This mouse isolate was chosen because it displays the ability to produce cytotoxin consistently and with the highest degree of cytotoxicity in the cell lines used in the cytotoxicity assays. In addition to trypticase soy broth (TSB), which is a standard media used to cultivate *K. oxytoca* and in which toxin production has been consistently observed, LB, a growth media with less nutrients compared to TSB, and Heart Brain Infusion (HBI) broth, which is richer in nutrients compared to TSB, were used. Toxin production under the three growth media was evaluated temporally. Toxin production was found to be highest in TSB, moderate in HBI, and lowest/nonexistence in LB media (Figure 3).

**Figure 4:**
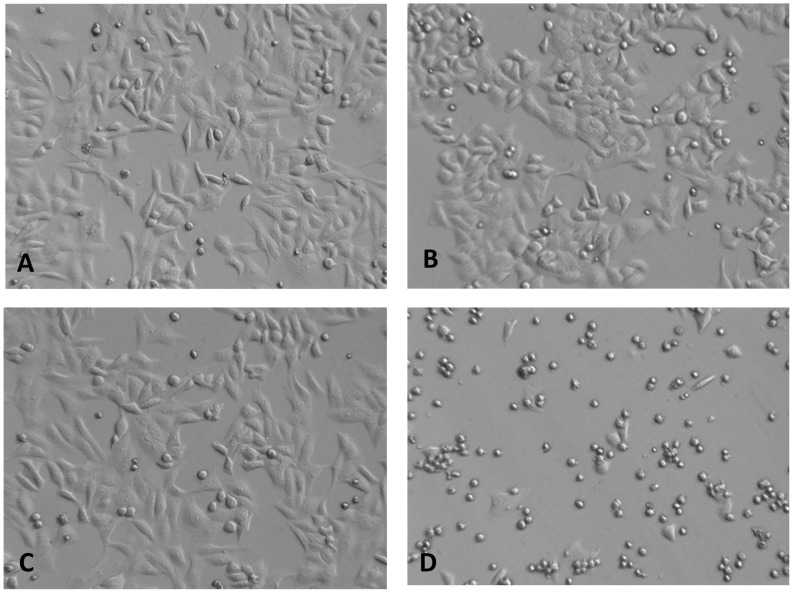
Soy components in TSB induced HEp-2 cell detachment and death. HEp-2 cells treated with **A)** media containing only casein extract (Tryptone; control); **B)** supernatant of *K. oxytoca*, 09-7231-1, grown in media containing only casein extract; **C)** media containing only soy extract (Soytone); **D)** supernatant of *K. oxytoca*, 09-7231-1, grown in the media containing only soy extract.

HBI contains nutrients mainly from animal products. In contrast, LB contains nutrient extracted from yeast. On the other hand, nutrients present in TSB are obtained mainly from soy product. Based on the observation that *K. oxytoca* grown in TSB produce supernatant with the highest toxicity, we hypothesize that soy product in TSB may contain compounds that contribute to the high toxin production in *K. oxytoca.* Therefore, we tested the cytotoxicity of supernatant obtained from three different media with well-defined source of nutrients: media contains soy extract only, media contains animal extract only (casein extract), and media contains both soy and casein extract (TSB). Media containing only the soy extract induces a high degree of cell abnormality and death leading to 1% confluency among HEp-2 cells as early as 18 hours post treatment (Figure 4). As expected, TSB media also induced cell abnormality and death in HEp-2, but to a lesser degree (30% confluency at 18 hours post treatment (data not shown)) compared to media containing only the soy extract. Like the negative control, media containing only the casein extract did not cause any significant alteration in monolayer confluency (∼100% confluency). All control media also did not have an effect on HEp-2 cells (data not shown), lessening the possibility that nutrients and components in the media are responsible for inducing HEp-2 cell death. Together, these results suggest that the soy component in TSB promotes cytotoxin production in *K.oxytoca*, 09-7231-1.

### *K. oxytoca* cytotoxin is a small heat labile molecule

It was previously demonstrated that human strains of *K. oxytoca* produced cytotoxin with an approximate size of 200 Daltons [22]. We investigated the size of cytotoxin produced by the mouse isolates, 09-7231-1, using protein size exclusion and *in vitro* cytotoxicity assays (See **Materials and Methods**). Both 3 KD concentrate and filtrate and 30 KD filtrate cause cell monolayer destruction resulting in <10% confluency, whereas 30 KD concentrate did not have obvious effects on the cell monolayer (∼100% confluency) (Figure S1). In addition, protease, trypsin, DNase, and RNase treatment did not alter the cytotoxic activity of the toxic molecule present in the supernatant of *K. oxytoca* (data not shown); however, heat treatment completely inactivated the cytotoxic activity of the toxic molecule (Figure S1). There results suggest that the cytotoxin present in the supernatant of *K. oxytoca*, 09-7231-2, is likely a small, heat labile compound. Furthermore, the compound is unlikely to be RNA, DNA, or protein. Additionally, supernatant placed in room temperature for as long as 8 weeks exhibited strong cytotoxicity on HEp-2 cells indicating that the toxin is stable at room temperature (data not shown).

### Tilivalline confers cytotoxicity *in vitro*

**Figure 5:**
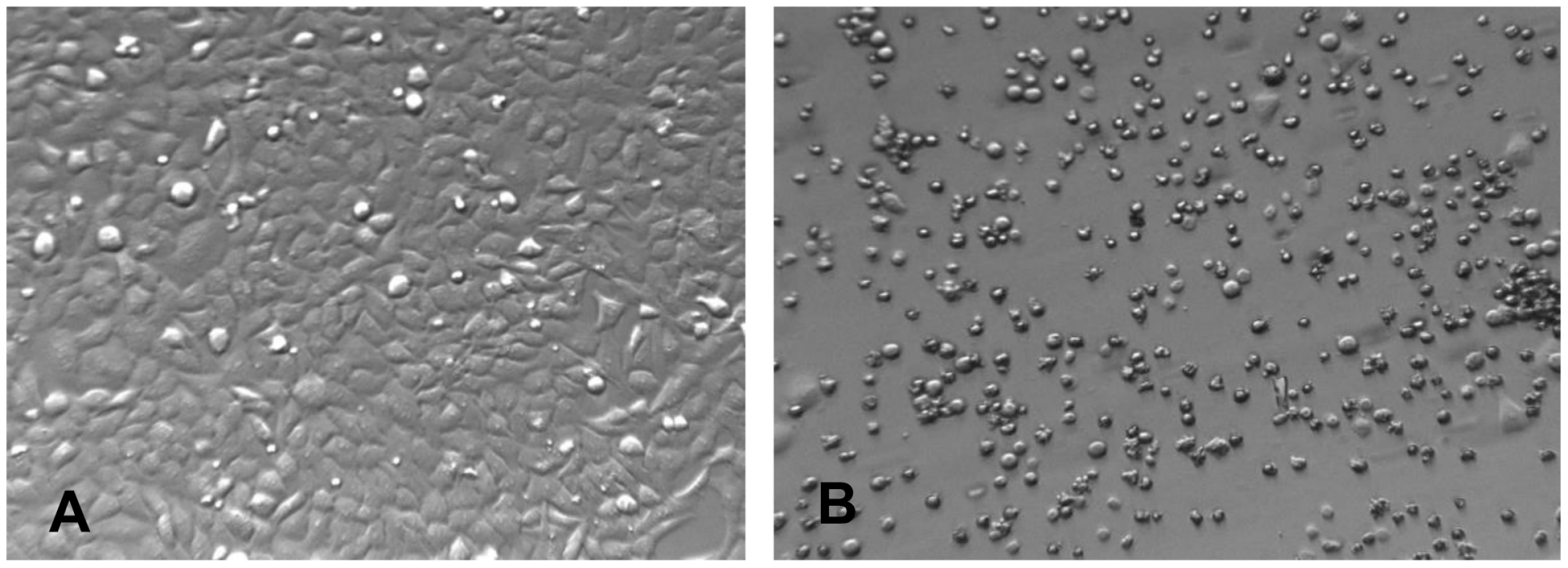
Tilivalline induced HEp-2 cell detachment and death. **A)** HEp-2 cells incubated with the control DMSO, which was used to dissolve tilivalline. The majority of cells adhered to the bottom of the cell culture well after 48 hours of culture; **B)** HEp-2 cells treated with purified tilivalline (1 µg/ml) were detached from the cell culture well after 48 hours of incubation.

Our studies suggested that the toxic compound was a small endogenous and heat labile molecule. Given previous studies from human *K. oxytoca* isolates indicated that tilivalline is the key toxin, we examined the cytotoxin-positive and cytotoxin-negative strain cultures for tilivalline. Comparison of LC-MS chromatograms from control and toxic culture media (Figure S2) revealed a compound in the toxic media with a protonated molecular ion ([M+ H^+^]^+^) at *m/z* =  334.1561, corresponding to a molecular weight of 333.1483 Da - within 2 ppm of that calculated for tilivalline: 333.1477 Da. CID spectra from the [M + H^+^]^+^ ion included fragments characteristic of tilivalline at *m/z* = 316.1454, 219.109, 199.1237, and 136.040 (Figure S3). The proton NMR spectrum (Figure S4) from a culture isolate, while weak due to the small amount of material, was also consistent with that reported for tilivalline [42]. Consistent with previous reports, tilivalline (1 µg/ml) induced cell abnormalities and death in HEp-2 cells (Figure 5).

### Toxin-induced perturbation using mRNA profiling

To investigate the molecular mechanism of toxin-induced cell death, we performed differential expression analyses on more than 34000 probes, each representing a gene, using the Human 4×44 GE v2 Agilent array. Plotting the log_2_ (fold change) and –log_10_ (corrected p-value) from the differential expression analysis, we found a large number of toxin-perturbed gene expression traits (see volcano plot in Figure S6, where each red point indicates a differentially expressed gene). Table S1 lists the most significantly perturbed genes (p_adjusted_<0.05) including the direction of effect. Notably, we found that the genes whose expression levels were significantly altered by the presence of the toxin were enriched in the following pathways: androgen receptor, hedgehog, ID signaling, NOTCH, TGFBR, and TNF alpha/NF-kB. Some of the most significant Gene Ontology functional annotations (Table S2 for comprehensive list) include *regulation of RNA metabolic process* (p_adjusted_ = 1.03×10^−35^), *regulation of nitrogen compound metabolic process* (p_adjusted_ = 1.46×10^−30^), *negative regulation of apoptosis* (p_adjusted_  = 1.2×10^−3^), *response to DNA damage stimulus* (p_adjusted_  = 1.75×10^−3^), and cell cycle (p_adjusted_  = 0.003).

### Potential virulence genes of *K. oxytoca*

Although the cytotoxic properties of tilivalline may in part explain the pathogenesis of the animal isolates *K. oxytoca*. other pathogenic mechanisms present in the bacteria may also contribute to its success as a pathogen. To further explore this possibility, the genome of a cytotoxin positive mouse isolate of *K. oxytoca*, 09-7231-1, was fully sequenced, and its genome was analyzed to determine if virulence genes identified in other bacteria were present. Based on current annotation, the entire genome of *K. oxytoca*, 09-7231-1, is 6.17Mb in size and has the G+C content of 54.78%. Nearly 88% (5,673 genes) of the genome is comprised of coding regions. Additionally, the genome contains 74 tRNAs and 22 rRNAs. Among genes annotated as having known function, genes that encode type I, type IV, and type VI secretion systems were detected. Other genes present in this strain include those involved in nitrate and allantoin metabolism, iron uptake, resistance to various toxic and antimicrobial compounds, e.g. beta-lactamase, macrolide, and citrate fermentation. A gene encoding ecotin, which is involved in host immune modulation, was also found. Evidence of transposon and phage genetic elements was also detected. Genes encoding type 1 and type 3 pilli shown to be involved in biofilm formation as well as a large set of housekeeping and structural genes were present (Table S3). These housekeeping and structural genes include those that are involved in the metabolism of various amino acids, fatty acids, sugars, as well as transportation of ion, organic and inorganic compounds, DNA synthesis, cell division and growth, stress responses, and motility. The genome also contains a gene encoding colicin, which promotes bacterial fitness by killing competing bacteria through membrane depolarization [43].

### Chromosomally encoded putative toxins

To investigate whether other known bacterial toxin(s) besides tilivalline may be produced by *K.oxytoca*, we performed *in silico* analyses using the blastp algorithm[44] against the whole genome assembly of *K. oxytoca*, 09-7231-1, that we generated. Comprehensive analysis of the genome sequence revealed genes with significant homology to toxin components of *Vibrio cholera, Citrobacter freundii, and Escherichia coli* (http://home.uchicago.edu/~egamazon/koxytoca/index.html). Our first round of BLAST analyses showed PaxB to have high homology (E-value = 0) to hypothetical proteins in the *K. oxytoca* strain. PaxB plays a role in secreting the RTX toxin, PaxA, in *Pasteurella aerogenes* [45]. Although the roles of these largely uncharacterized *K. oxytoca* genes have not been fully elucidated, the high homology between the proteins suggests some type of transportation role that warrants future functional validation.

**Figure 6:**
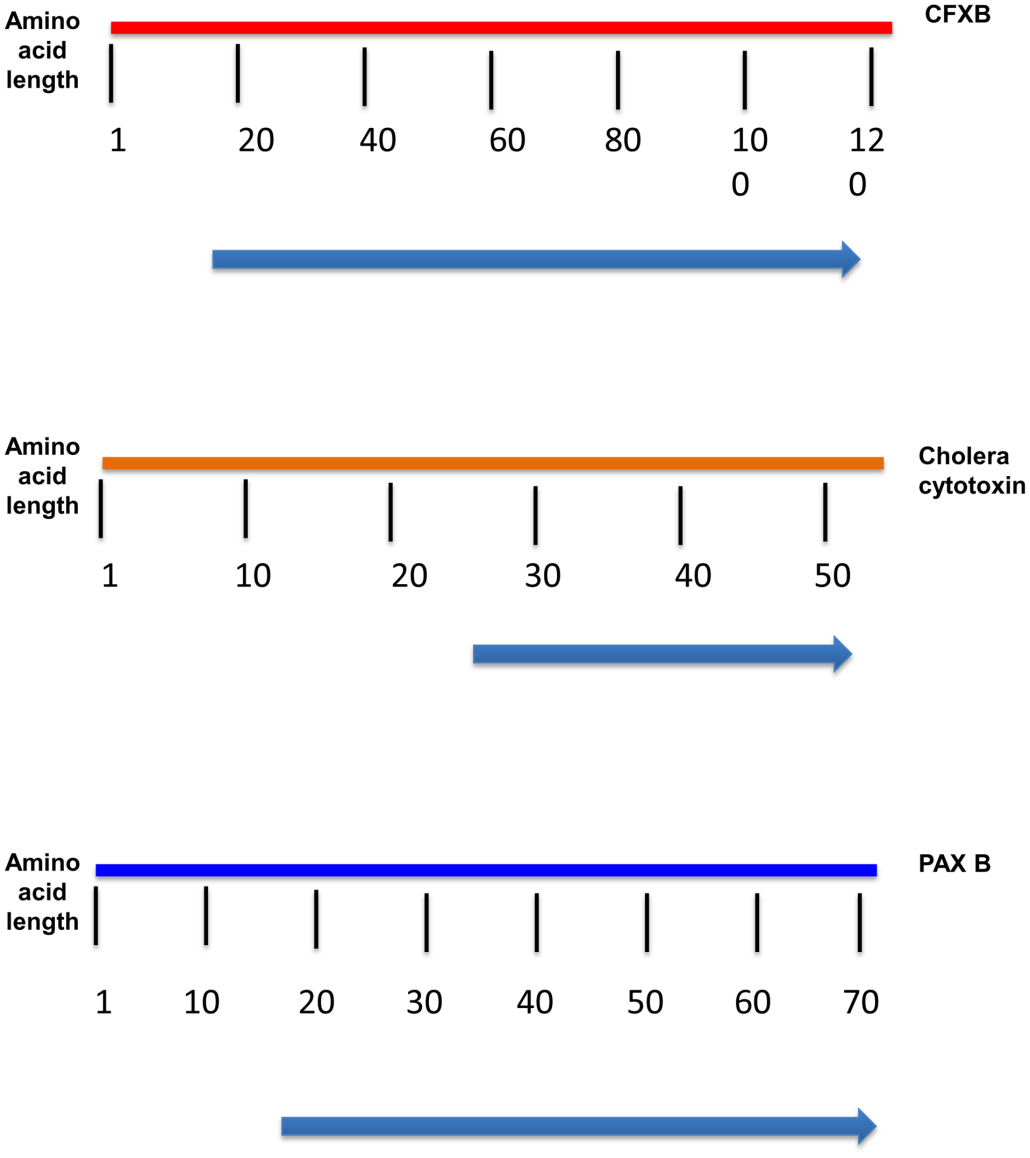
The reference proteins encoded by CTA, CFX B, and PAX B are displayed as colored bars. The arrow beneath the bar shows the region in the reference protein to which the corresponding *K. Oxytoca* protein aligns from the BLAST analysis.

Additionally, genes encoding proteins with high similarity to known heat-labile toxins in other enteric pathogens were identified (Figure 6). Two of these toxins - the cholera toxin (CT) in *V. cholerae*, and the heat-labile toxin (LT) in enterotoxigenic *E. coli* strains (ETEC) - are particularly interesting. They both typically consist of 2 subunits—a catalytic A subunit and an immunogenic B subunit [46]. BLAST analysis indicated that the *K. oxytoca* strain contained a gene with homology to a partial sequence of a known cholera toxin (CT) A subunit, containing the conserved domain pfam01375 (Figure 6).

BLAST analysis also revealed genes with homology (E-value<0.25) to CFXB from *C. freundii* 48. CFXB encodes a 12-residue polypeptide with 73.8% sequence identity with the beta subunits of LT and 72.8% sequence identity with the beta subunit of CT [46]. CFXB, with a molecular weight of 14.2 kDa, contains the conserved domain pfam01376 for the heat-labile enterotoxin beta chain.

## Discussion

This study investigated the possibility that animal isolates of *K. oxytoca* produce a specific cytotoxin and explored the mechanisms governing its pathogenicity with *in vitro* assays as well as genome sequencing and analysis. To accomplish this, we characterized 64 isolates of *K. oxytoca* from various animal species. Of these *K. oxytoca* strains, 18 (28%) produced supernatant that caused HEp-2 and HeLa cell detachment and death when grown under *in vitro* conditions previously used to identify cytotoxin-positive human strains of *K. oxytoca* [4], [22], [23]. This indicates that *K. oxytoca* of animals, similar to those isolated from humans, produce cytotoxin that, in part, may be responsible for promoting *K. oxytoca* pathogenesis.

Our results indicate *K. oxytoca* cytotoxin production is under strict environmental regulation. Specifically, microaerobic and aerobic conditions i.e, AG and MG, were more effective in promoting cytotoxin production among *K. oxytoca* strains investigated compared to anaerobic (AN) and static aerobic conditions (AR). Although AR provides anaerobic condition initially, it actually resembles an anaerobic condition after 16 hours of culture when oxygen is depleted by the growing population of bacteria. The resulting anaerobic condition of AR may explain the lower effect on cytotoxin production similarly observed in AN as compared to AG and MG. The observation that AG and MG have greater promoting effects on cytotoxin production in *K. oxytoca* compared to AN may, in part, explain why *K. oxytoca* has been reported to cause lesions in the mammary gland, eye, ear, respiratory and urogenital tract, where O_2_ tensions are relatively high, and diseases associated with gastrointestinal tract, which offers *K. oxytoca* a microaerobic environment for growth. The aerobic and microaerobic conditions these tissues provide may favor high level of cytotoxin production and thus greater cell death. These results are not surprising as environmental regulation of virulence genes and toxin production has also been observed in other gastrointestinal pathogens, such as *Shigella* spp., *E. coli, Yersinia enterocolitica* and *Mycobacterium avium* [37], [38], [39]. Surprisingly, cytotoxicity of *K. oxytoca* was also found to be influenced by nutrients, and soy extract appears to have promoting effects on the production of toxin. It is increasingly cited that soy products have a negative effect on gastrointestinal health in animals and humans. For example, there is an increasing incidence of food protein-induced enterocolitis and other inflammatory bowel disease entities that coincide with increased consumption of soy products [47], [48], [49]. It is therefore possible that soy-induced toxin production in gut microbes, including *K. oxytoca*, may in part play a role in the pathogenesis of inflammatory bowel diseases in susceptible individuals. Further investigations are needed to investigate this possibility and to determine how soy products promote cytotoxin production in *K. oxytoca.*

Of the 4 clinical *K. oxytoca* isolates obtained from laboratory animals, only one isolate was cytotoxin positive. In a related study, *K. oxytoca* was isolated from 14 of 200 patients (7%) being screened for vancomycin-resistant enterococci (VRE), but only one of the 14 isolates (7.1%) was cytotoxic [12]. In another study, *K. oxytoca* was isolated from 42 of 429 patients (9.8%) tested for *C. difficile* cytotoxicity; only 10 isolates (23.8%) were cytotoxic [12]. It is likely that the low percentage of cytotoxin production among the clinical isolates of *K. oxytoca* in both humans and animals is due to the insensitivity of the current *in vitro* assays that do not provide appropriate environmental cues necessary to induce cytotoxin production in this bacterium. As demonstrated by our results, cytotoxin production in *K. oxytoca* appears to be under strict environmental regulation. Identifying environmental signals that induce cytotoxin production in *K. oxytoca* may provide additional insights into *K. oxytoca* pathogenesis and possible therapeutic strategies.

The majority of mouse isolates of *K. oxytoca* in this study were cultured from the feces or respiratory tract of sentinel rodents during routine health screening. This suggests that the respiratory and gastrointestinal tract may be the preferred site of colonization by *K. oxytoca* in mice. However, the fact that a few isolates of *K. oxytoca* were isolated from other tissues, including palpebral conjunctiva, tumor, and, mammary gland, indicates that *K. oxytoca* can also colonize other organs.

Antibiotic resistance patterns appear to vary considerably among different strains of *K. oxytoca*. Strains of *K. oxytoca* are resistant to amino-penicillins and carboxy-penicillins due to the production of β-lactamases. Antimicrobial susceptibility testing indicated that all of the *K. oxytoca* strains investigated were resistant to ampicillin, suggesting these isolates of *K.oxytoca* are able to synthesize B-lactamases. This is consistent with the presence of beta-lactamases that encode a gene present in the genome of *K. oxytoca* 09-7231. Resistance to amoxicillin/clavuranate was also observed in select strains of *K. oxytoca*. Interestingly, *K. oxytoca* isolates originating from non-human primates showed resistance not only to ampicillin, cephalothin and amoxicillin/clavuranate, but also to trimethoprim/sulfamethoxazole, enrofloxacin, and gentamicin. The high percentage of antibiotic-resistant strains of *K. oxytoca* isolated from nonhuman primates may indicate the liberal use of antibiotics in primates, particularly when the animals are in quarantine and placed on prophylactic antibiotics prior to release for research purposes [30].

Genomic analysis of a cytotoxin-positive *K. oxytoca* isolated from a clinically affected mouse revealed homologs of multiple genes that promote pathogenicity in other pathogens. These include type I, type IV, and type VI secretion systems. The type IV secretion system promotes pathogenicity in *H. pylori* through injection of cagA [50]. More recently, the type VI secretion system has been demonstrated to promote bacterial competition, host cell adhesion, and invasion in *Escherichia coli*, *Campylobacter jejuni*, *Salmonella* spp [51], [52]. In clinical isolates of *K. pneumoniae*, cultured from a liver abscess in a human, genes involved in allantoin metabolism were highly and uniquely upregulated, while the non-clinical strain of *K. pneumoniae* lacked genes involved in this metabolic pathway[53]. The mouse clinical isolate of *K. oxytoca* investigated in this study also contains a large number of genes involved in allantoin metabolism, including *alls* and *allr*, which were found in the pathogenic strain of *K. pneumoniae*. This suggests that, like *K. pneumoniae* [53], *K. oxytoca* may utilize allantoin metabolism to initiate extraintestinal pathology, particularly in a pathological condition when high allantoin concentration in the tissues is present, while other nitrogen energy sources are limited [53]. The *K. oxytoca* isolate also contains genes involved in citrate fermentation, previously found in some clinical isolates of *K. pneumoniae*, as well as genes encoding ecotin. The presence of ecotin, a protease inhibitor present in *Yersinia pestis*, *E. coli* and *Pseudomonas aeruginosa*, known to inhibit neutrophil elastase, suggests that *K. oxytoca* may utilize this protein to manipulate neutrophil function, thereby aiding the persistence of the organism[54]. Indeed, *K. oxytoca* shows a remarkable ability to persist in the gastrointestinal tract even after aggressive antibiotic therapy with antibiotics that appear to be effective against other resident bacteria [4]. *In silico* analysis also reveals three genes present in the genome of *K. oxytoca* that show partial homology to known toxins and toxin transporters previously identified in other pathogenic bacteria, including PAXA, cholera toxin, and CFXB. Further investigation is needed to determine the biological relevance of these toxin homologs in *K. oxytoca* pathogenesis.

Initial description of a low-molecular-weight (∼217 Da) cytotoxin, produced by cultured strains of *K. oxytoca* isolated from humans with hemorrhagic diarrhea, was reported by Minami and co-workers, although its structure was not characterized [22], [23]. Mohr and Budzikiewicz had earlier identified a low molecular-weight (333 Da) cytotoxic compound, produced by *K. pneumoniae* serova *oxytoca*, as tilivalline [24]. Here we demonstrate that cultured animal isolates of *K. oxytoca* also produce high levels of tilivalline. The purified compound was tested *in vitro* for cytotoxic properties and was found to induce cell abnormality and death in HEp-2 cells confirming its cytotoxic property.

Previous studies to determine the molecular mechanisms of toxin induced cell death in *K. oxytoca* suggest that the mechanism is primarily through inhibition of DNA synthesis [22], [23]. Our whole transcriptomic profiling reveals that tilivalline induced global perturbation of genes involved in numerous fundamental cellular processes, including cell division, nitrogen metabolism, RNA metabolic processing, DNA damage repair processes, apoptosis, and androgen signaling, as early as 6 hours after HEp-2 cells were exposed to the toxin when cell abnormalities are not yet observed. Although the primary target of tilivalline may be in the synthesis of DNA, our transcriptome data highlight the possibility of simultaneous effects of tilivalline on multiple cellular processes and pathways. Further investigations are required to determine the exact mechanism of tilivalline-induced cell death.

In conclusion, this study provides evidence that - similar to *K. oxytoca* strains in humans - animal isolates of *K. oxytoca* are capable of producing cytotoxin *in vitro*. The roles that *K. oxytoca* cytotoxin play in induction of genitourinary diseases or abscesses in other tissues in mice and its pathogenic potential in other animals remain to be defined.

## Supporting Information

Figure S1 **Cytotoxicity of supernatant on HEp-2 cells subjected to A) 30K concentrate; B) 30 K filtrate; C) 3K concentrate; D) 3K filtrate; E) heat-treated supernatant of** ***K. oxytoca,*** **09-7231-1; F) supernatant of K.** ***oxytoca*** **, 09-7231-1, without heat treatment.** Note the low confluency of B), C), D), and F) suggesting strong cytotoxic activity.
(TIF)

Figure S2 **LC/MS total-ion chromatograms.** **A**) soy broth extract; **B**) soy broth extract from 9-hour toxic culture; **C**) soy broth extract from 24-hour toxic culture; **D**) soy broth extract from 16-hour culture of negative strain (non-toxic). Inset: background-subtracted mass spectrum of the compound eluting near 2.5 minutes.
(TIF)

Figure S3 **MS/MS spectrum from m/z 334.156 (collision energy  =  20 V), with suggested fragment structures.**
(TIF)

Figure S4 **^1^H NMR spectrum.** Recorded on a Varian Inova-500 instrument operating at 500.13 MHz using 5 mm O.D. thin-walled precision NMR tubes (Wilmad). Chemical shifts are relative to pyridine-*d_5_* using Varian 5 mm PFG-probes at 22°C.
(TIF)

Figure S5 **HPLC chromatogram with UV/vis detection for the isolation oft tilivalline (24.3 min).**
(TIF)

Figure S6 **A volcano plot illustrated differentially regulated genes.**
(TIF)

Table S1 **The most significantly perturbed genes (adjusted p-value <0.05).** In addition to the p-value from the differential expression analysis, log-fold change and direction of effect are shown.
(XLSX)

Table S2 **The most significant Gene Ontology functional annotations.** The most significant functional annotations implicated by the differentially expressed genes include *regulation of RNA metabolic process* (p_adjusted_ = 1.03×10^−35^), *regulation of nitrogen compound metabolic process* (p_adjusted_ = 1.46×10^−30^), *negative regulation of apoptosis* (p_adjusted_ = 1.2×10^−3^), *response to DNA damage stimulus* (p_adjusted_ = 1.75×10^−3^), and cell cycle (p_adjusted_ = 0.003).
(XLSX)

Table S3 **Potential virulence genes found in K. oxytoca, 09-7231-1".**
(DOCX)

## References

[1] Gorkiewicz G (2009) Nosocomial and antibiotic-associated diarrhea caused by organisms other than *Clostridium difficile* . Int J Antimicrob Agents 33 Suppl 1S37–41. 19303568 10.1016/S0924-8579(09)70015-9

[2] Podschun R, Ullmann U (1998) *Klebsiella* spp. as nosocomial pathogens: epidemiology, taxonomy, typing methods, and pathogenicity factors. Clin Microbiol Rev 11: 589–603. 9767057 10.1128/cmr.11.4.589 PMC88898

[3] Savino F, Cordisco L, Tarasco V, Calabrese R, Palumeri E, et al (2009) Molecular identification of coliform bacteria from colicky breastfed infants. Acta Paediatr 98: 1582–1588. 19604166 10.1111/j.1651-2227.2009.01419.x

[4] Hoffmann KM, Deutschmann A, Weitzer C, Joainig M, Zechner E, et al (2010) Antibiotic-associated hemorrhagic colitis caused by cytotoxin-producing *Klebsiella oxytoca* . Pediatrics 125: e960–963. 20194278 10.1542/peds.2009-1751

[5] Savino F, Cordisco L, Tarasco V, Locatelli E, Di Gioia D, et al (2011) Antagonistic effect of *Lactobacillus* strains against gas-producing coliforms isolated from colicky infants. BMC Microbiol 11: 157. 21718486 10.1186/1471-2180-11-157 PMC3224137

[6] Murphy MS (2008) Management of bloody diarrhea in children in primary care. BMJ 336: 1010–1015. 18456632 10.1136/bmj.39542.440417.BE PMC2364807

[7] Hogenauer C, Langner C, Beubler E, Lippe IT, Schicho R, et al (2006) *Klebsiella oxytoca* as a causative organism of antibiotic-associated hemorrhagic colitis. N Engl J Med 355: 2418–2426. 17151365 10.1056/NEJMoa054765

[8] Zollner-Schwetz I, Hogenauer C, Joainig M, Weberhofer P, Gorkiewicz G, et al (2008) Role of *Klebsiella oxytoca* in antibiotic-associated diarrhea. Clinical infectious diseases: an official publication of the Infectious Diseases Society of America 47: e74–78. 18808355 10.1086/592074

[9] Al-Anazi KA, Al-Jasser AM, Al-Zahrani HA, Chaudhri N, Al-Mohareb FI (2008) Klebsiella oxytoca bacteremia causing septic shock in recipients of hematopoietic stem cell transplant: Two case reports. Cases J 1: 160. 18801186 10.1186/1757-1626-1-160 PMC2556667

[10] Lin RD, Hsueh PR, Chang SC, Chen YC, Hsieh WC, et al (1997) Bacteremia due to Klebsiella oxytoca: clinical features of patients and antimicrobial susceptibilities of the isolates. Clinical infectious diseases: an official publication of the Infectious Diseases Society of America 24: 1217–1222. 9195086 10.1086/513637

[11] Menard A, Harambat J, Pereyre S, Pontailler JR, Megraud F, et al (2010) First report of septic arthritis caused by Klebsiella oxytoca. J Clin Microbiol 48: 3021–3023. 20573877 10.1128/JCM.00302-10 PMC2916600

[12] Smith SA, Campbell SJ, Webster D, Curley M, Leddin D, et al (2009) A study of the prevalence of cytotoxic and non-cytotoxic *Klebsiella oxytoca* fecal colonization in two patient populations. Can J Infect Dis Med Microbiol 20: e169–172. 21119796 10.1155/2009/913895 PMC2807256

[13] Sorli L, Miro E, Segura C, Navarro F, Grau S, et al (2011) Intra- and inter-species spread of carbapenemase genes in a non-hospitalized patient. Eur J Clin Microbiol Infect Dis 30: 1551–1555. 21491175 10.1007/s10096-011-1259-1

[14] Youssef D, Shams W, Kareem Abu Malouh A, Al-Abbadi MA (2012) Chronic organizing retroperitoneal abscess caused by Klebsiella oxytoca masquerading as sarcoma: recognition by Diff-Quik stain on FNA material. Diagn Cytopathol 40: 747–750. 21538961 10.1002/dc.21701

[15] Zarate MS, Gales AC, Picao RC, Pujol GS, Lanza A, et al (2008) Outbreak of OXY-2-Producing Klebsiella oxytoca in a renal transplant unit. J Clin Microbiol 46: 2099–2101. 18417660 10.1128/JCM.00194-08 PMC2446844

[16] Sanchez E, Donat E, Ribes-Koninckx C, Fernandez-Murga L, Sanz Y (2013) Duodenal-mucosal bacteria associated with celiac disease in children. Appl Environ Microbiol. 10.1128/AEM.00869-13 PMC3754165 23835180

[17] Davis JK, Gaertner DJ, Cox NR, Lindsey JR, Cassell GH, et al (1987) The role of *Klebsiella oxytoca* in utero-ovarian infection of B6C3F1 mice. Lab Anim Sci 37: 159–166. 3599883

[18] Rao GN, Hickman RL, Seilkop SK, Boorman GA (1987) Utero-ovarian infection in aged B6C3F1 mice. Lab Anim Sci 37: 153–158. 3298846

[19] Bleich A, Kirsch P, Sahly H, Fahey J, Smoczek A, et al (2008) *Klebsiella oxytoca*: opportunistic infections in laboratory rodents. Lab Anim 42: 369–375. 18625592 10.1258/la.2007.06026e

[20] Foreman O, Kavirayani AM, Griffey SM, Reader R, Shultz LD (2011) Opportunistic bacterial infections in breeding colonies of the NSG mouse strain. Vet Pathol 48: 495–499. 20817888 10.1177/0300985810378282 PMC3101569

[21] Nemet Z, Szenci O, Horvath A, Makrai L, Kis T, et al (2011) Outbreak of *Klebsiella oxytoca* enterocolitis on a rabbit farm in Hungary. The Veterinary record 168: 243. 10.1136/vr.c6350 21493575

[22] Minami J, Okabe A, Shiode J, Hayashi H (1989) Production of a unique cytotoxin by *Klebsiella oxytoca* . Microb Pathog 7: 203–211. 2615635 10.1016/0882-4010(89)90056-9

[23] Minami J, Saito S, Yoshida T, Uemura T, Okabe A (1992) Biological activities and chemical composition of a cytotoxin of *Klebsiella oxytoca* . J Gen Microbiol 138: 1921–1927. 1402792 10.1099/00221287-138-9-1921

[24] Mohr N, Budzikiewicz H (1982) Tilivalline, a new pyrrolo[2,1-*c*][1,4] benzodiazepine metabolite from *klebsiella* . Tetrahedron 38: 147–152.

[25] Kohda K, Yamagami N, Kasamatsu T, Aoyama T, Shioiri T (1995) Wide difference between the cytotoxicity of the 11-alpha-and 11-beta-cyano analogues of tilivalline and their epimeric conversion. Biochemical Pharmacology 49: 1063–1068. 7748186 10.1016/0006-2952(95)98502-z

[26] Shioiri T, Aoyama T, Yamagami N, Shimizu N, Mori N, et al (1995) Structure-cytotoxicity relationship of tilivalline derivatives. Anti-cancer drug design 10: 167–176. 7710637

[27] Joainig MM, Gorkiewicz G, Leitner E, Weberhofer P, Zollner-Schwetz I, et al (2010) Cytotoxic effects of *Klebsiella oxytoca* strains isolated from patients with antibiotic-associated hemorrhagic colitis or other diseases caused by infections and from healthy subjects. J Clin Microbiol 48: 817–824. 20053860 10.1128/JCM.01741-09 PMC2832427

[28] Kovtunovych G, Lytvynenko T, Negrutska V, Lar O, Brisse S, et al (2003) Identification of *Klebsiella oxytoca* using a specific PCR assay targeting the polygalacturonase pehX gene. Res Microbiol 154: 587–592. 14527660 10.1016/S0923-2508(03)00148-7

[29] Fox JG, Shen Z, Muthupalani S, Rogers AR, Kirchain SM, et al (2009) Chronic hepatitis, hepatic dysplasia, fibrosis, and biliary hyperplasia in hamsters naturally infected with a novel Helicobacter classified in the H. bilis cluster. J Clin Microbiol 47: 3673–3681. 19759229 10.1128/JCM.00879-09 PMC2772605

[30] Fox JG, Handt L, Xu S, Shen Z, Dewhirst FE, et al (2001) Novel *Helicobacter* species isolated from rhesus monkeys with chronic idiopathic colitis. J Med Microbiol 50: 421–429. 11339249 10.1099/0022-1317-50-5-421

[31] Zheng J, Meng J, Zhao S, Singh R, Song W (2008) Campylobacter-induced interleukin-8 secretion in polarized human intestinal epithelial cells requires Campylobacter-secreted cytolethal distending toxin- and Toll-like receptor-mediated activation of NF-kappaB. Infect Immun 76: 4498–4508. 18644884 10.1128/IAI.01317-07 PMC2546826

[32] Chien CC, Taylor NS, Ge Z, Schauer DB, Young VB, et al (2000) Identification of cdtB homologues and cytolethal distending toxin activity in enterohepatic *Helicobacter* spp. J Med Microbiol 49: 525–534. 10847206 10.1099/0022-1317-49-6-525

[33] Henikoff S, Henikoff JG (1992) Amino acid substitution matrices from protein blocks. Proc Natl Acad Sci U S A 89: 10915–10919. 1438297 10.1073/pnas.89.22.10915 PMC50453

[34] Bateman A, Birney E, Cerruti L, Durbin R, Etwiller L, et al (2002) The Pfam protein families database. Nucleic Acids Res 30: 276–280. 11752314 10.1093/nar/30.1.276 PMC99071

[35] Boekhorst J, Snel B (2007) Identification of homologs in insignificant blast hits by exploiting extrinsic gene properties. BMC Bioinformatics 8: 356. 17888146 10.1186/1471-2105-8-356 PMC2048517

[36] Gasteiger E, Hoogland C, Gattiker A, Duvaud S, Wilkins MR, et al. (2005) The Proteomics Protocols Handbook. Totowa, N.J.: Humana Press. xviii, 988 p. p.

[37] Bermudez LE, Petrofsky M, Goodman J (1997) Exposure to low oxygen tension and increased osmolarity enhance the ability of *Mycobacterium avium* to enter intestinal epithelial (HT-29) cells. Infect Immun 65: 3768–3773. 9284150 10.1128/iai.65.9.3768-3773.1997 PMC175537

[38] Diard S, Toribio AL, Boum Y, Vigier F, Kansau I, et al (2006) Environmental signals implicated in Dr fimbriae release by pathogenic *Escherichia coli* . Microbes Infect 8: 1851–1858. 16815721 10.1016/j.micinf.2006.02.023

[39] Marteyn B, West NP, Browning DF, Cole JA, Shaw JG, et al (2010) Modulation of *Shigella* virulence in response to available oxygen *in vivo* . Nature 465: 355–358. 20436458 10.1038/nature08970 PMC3750455

[40] Pederson KJ, Pierson DE (1995) Ail expression in *Yersinia enterocolitica* is affected by oxygen tension. Infect Immun 63: 4199–4201. 7558344 10.1128/iai.63.10.4199-4201.1995 PMC173595

[41] Lei XH, Bochner BR (2013) Using phenotype microarrays to determine culture conditions that induce or repress toxin production by *Clostridium difficile* and other microorganisms. PLoS One 8: e56545. 23437164 10.1371/journal.pone.0056545 PMC3577869

[42] Nagasaka T, Koseki Y (1998) Stereoselective Synthesis of Tilivalline(1). The Journal of organic chemistry 63: 6797–6801. 11672297 10.1021/jo972158g

[43] Johnson CL, Ridley H, Pengelly RJ, Salleh MZ, Lakey JH (2013) The unstructured domain of colicin N kills *Escherichia coli* . Mol Microbiol 89: 84–95. 23672584 10.1111/mmi.12260 PMC3739937

[44] Altschul SF, Gish W, Miller W, Myers EW, Lipman DJ (1990) Basic local alignment search tool. J Mol Biol 215: 403–410. 2231712 10.1016/S0022-2836(05)80360-2

[45] Kuhnert P, Heyberger-Meyer B, Nicolet J, Frey J (2000) Characterization of PaxA and its operon: a cohemolytic RTX toxin determinant from pathogenic *Pasteurella aerogenes* . Infect Immun 68: 6–12. 10603361 10.1128/iai.68.1.6-12.2000 PMC97094

[46] Karasawa T, Ito H, Tsukamoto T, Yamasaki S, Kurazono H, et al (2002) Cloning and characterization of genes encoding homologues of the B subunit of cholera toxin and the *Escherichia coli* heat-labile enterotoxin from clinical isolates of *Citrobacter freundii* and *E. coli* . Infect Immun 70: 7153–7155. 12438400 10.1128/IAI.70.12.7153-7155.2002 PMC133046

[47] Hedrera MI, Galdames JA, Jimenez-Reyes MF, Reyes AE, Avendano-Herrera R, et al (2013) Soybean meal induces intestinal inflammation in zebrafish larvae. PLoS One 8: e69983. 23894568 10.1371/journal.pone.0069983 PMC3720926

[48] Nowak-Wegrzyn A, Muraro A (2009) Food protein-induced enterocolitis syndrome. Curr Opin Allergy Clin Immunol 9: 371–377. 19474706 10.1097/ACI.0b013e32832d6315

[49] Leonard SA, Nowak-Wegrzyn A (2013) Manifestations, diagnosis, and management of food protein-induced enterocolitis syndrome. Pediatr Ann 42: 135–140. 23805961 10.3928/00904481-20130619-11

[50] Muller A (2012) Multistep activation of the *Helicobacter pylori* effector CagA. J Clin Invest 122: 1192–1195. 22378039 10.1172/JCI61578 PMC3314475

[51] Coulthurst SJ (2013) The Type VI secretion system - a widespread and versatile cell targeting system. Res Microbiol 164: 640–654. 23542428 10.1016/j.resmic.2013.03.017

[52] Lertpiriyapong K, Gamazon ER, Feng Y, Park DS, Pang J, et al (2012) *Campylobacter jejuni* type VI secretion system: roles in adaptation to deoxycholic acid, host cell adherence, invasion, and in vivo colonization. PLoS One 7: e42842. 22952616 10.1371/journal.pone.0042842 PMC3428339

[53] Chou HC, Lee CZ, Ma LC, Fang CT, Chang SC, et al (2004) Isolation of a chromosomal region of *Klebsiella pneumoniae* associated with allantoin metabolism and liver infection. Infect Immun 72: 3783–3792. 15213119 10.1128/IAI.72.7.3783-3792.2004 PMC427404

[54] Eggers CT, Murray IA, Delmar VA, Day AG, Craik CS (2004) The periplasmic serine protease inhibitor ecotin protects bacteria against neutrophil elastase. Biochem J 379: 107–118. 14705961 10.1042/BJ20031790 PMC1224055

