# Supplementary figures and images for: Cytotoxic and Pathogenic Properties of Klebsiella oxytoca Isolated from Laboratory Animals
Source: PLoS One. 2014 Jul 24;9(7):e100542. doi: 10.1371/journal.pone.0100542 (PMC4109914; doi:10.1371/journal.pone.0100542)

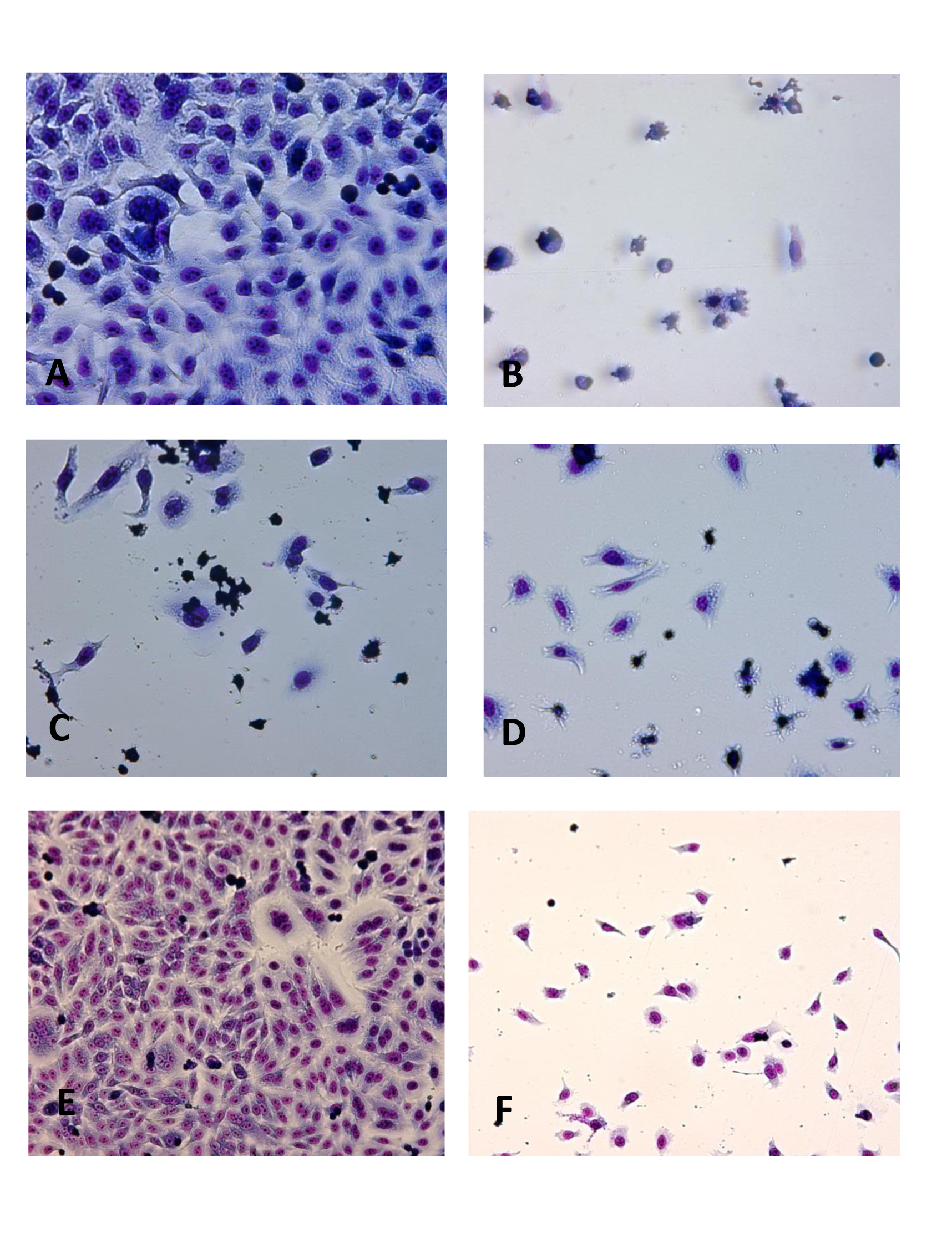

Supplement: Figure S1 — Cytotoxicity of supernatant on HEp-2 cells subjected to A) 30K concentrate; B) 30 K filtrate; C) 3K concentrate; D) 3K filtrate; E) heat-treated supernatant of K. oxytoca, 09-7231-1; F) supernatant of K. oxytoca , 09-7231-1, without heat treatment. Note the low confluency of B), C), D), and F) suggesting strong cytotoxic activity. (TIF) [file pone.0100542.s001.tif]

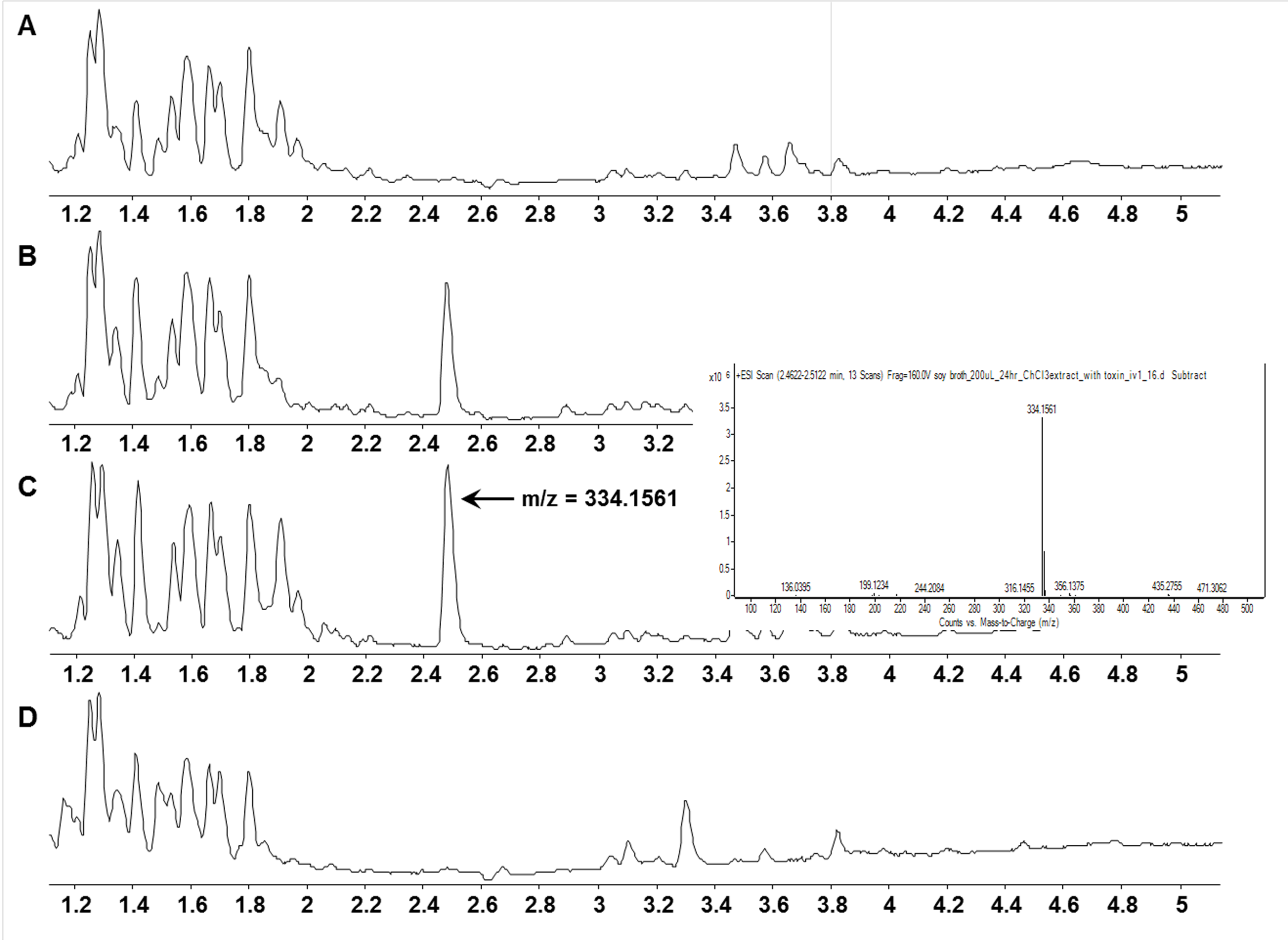

Supplement: Figure S2 — LC/MS total-ion chromatograms. A) soy broth extract; B) soy broth extract from 9-hour toxic culture; C) soy broth extract from 24-hour toxic culture; D) soy broth extract from 16-hour culture of negative strain (non-toxic). Inset: background-subtracted mass spectrum of the compound eluting near 2.5 minutes. (TIF) [file pone.0100542.s002.tif]

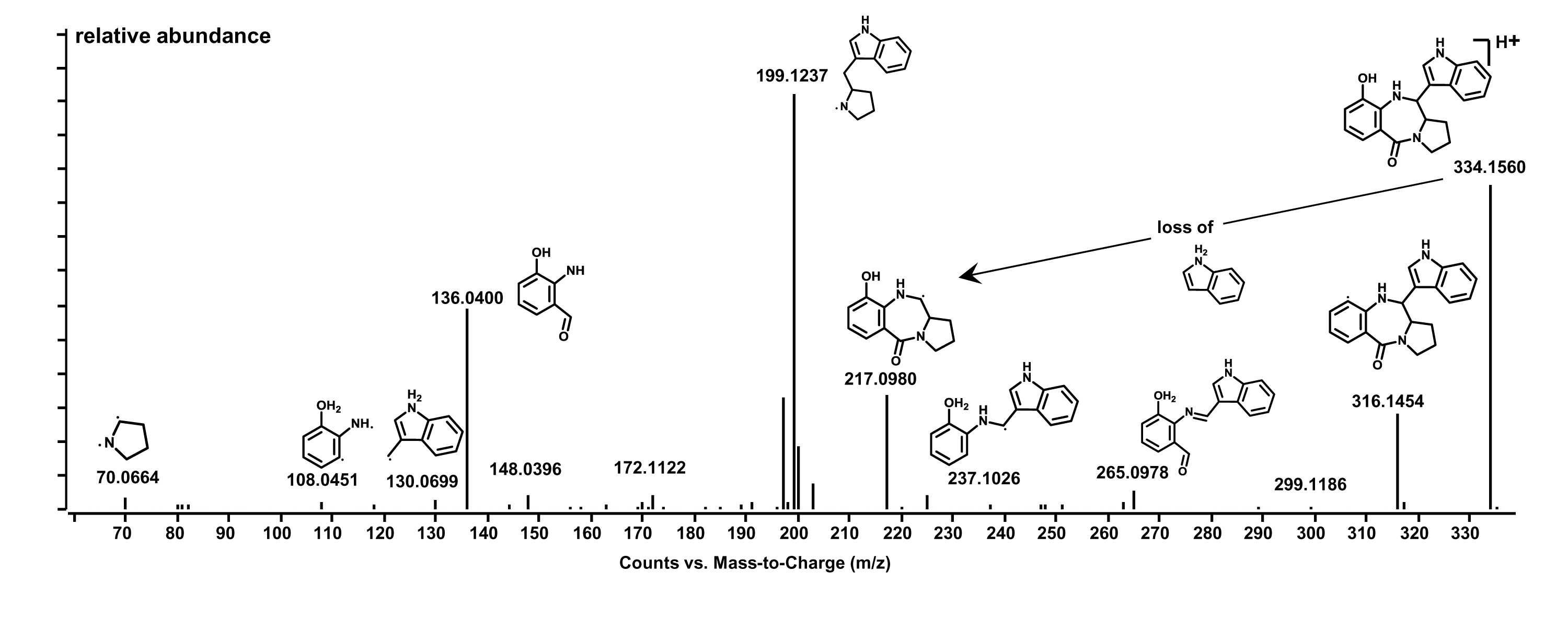

Supplement: Figure S3 — MS/MS spectrum from m/z 334.156 (collision energy = 20 V), with suggested fragment structures. (TIF) [file pone.0100542.s003.tif]

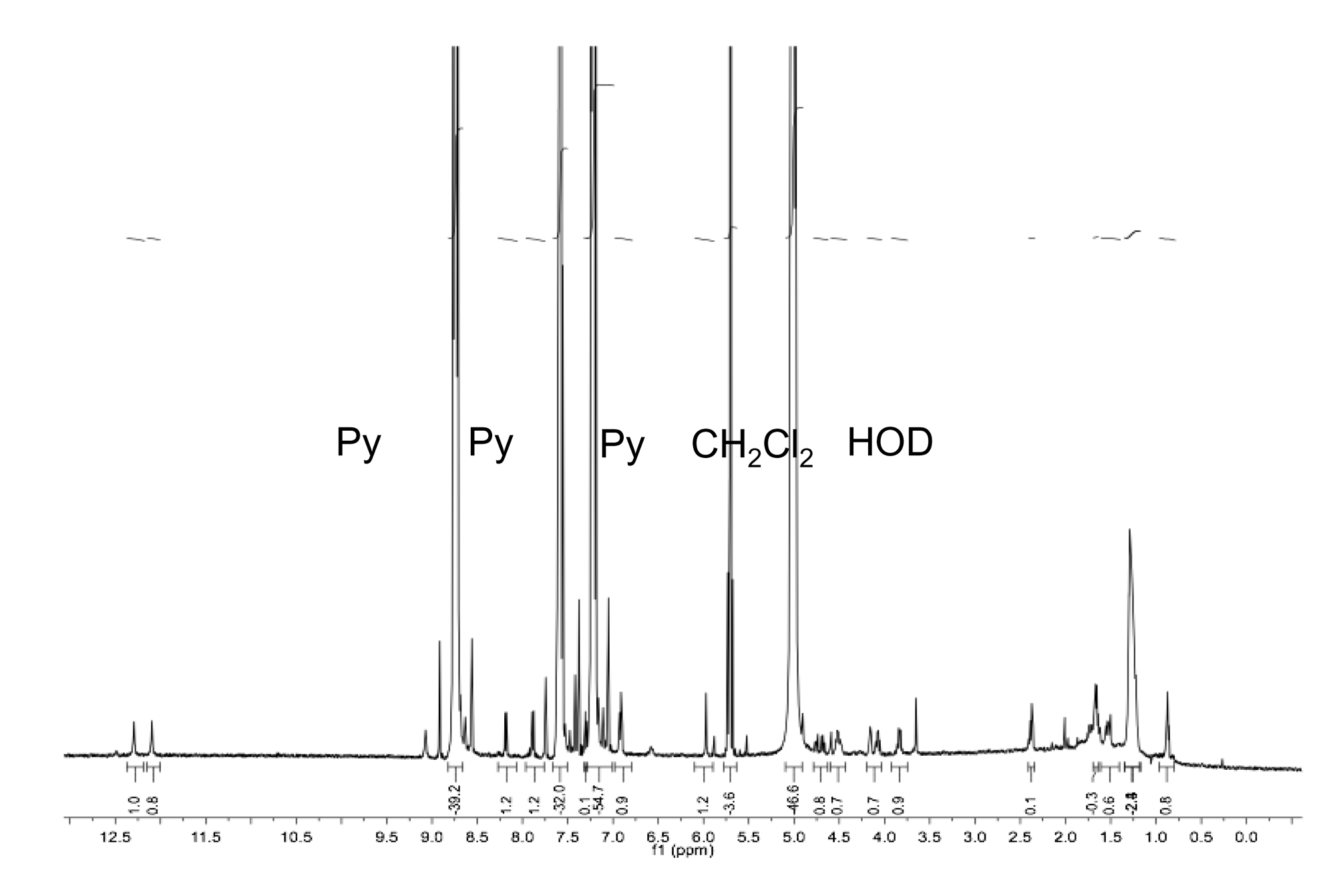

Supplement: Figure S4 — 1H NMR spectrum. Recorded on a Varian Inova-500 instrument operating at 500.13 MHz using 5 mm O.D. thin-walled precision NMR tubes (Wilmad). Chemical shifts are relative to pyridine-d5 using Varian 5 mm PFG-probes at 22°C. (TIF) [file pone.0100542.s004.tif]

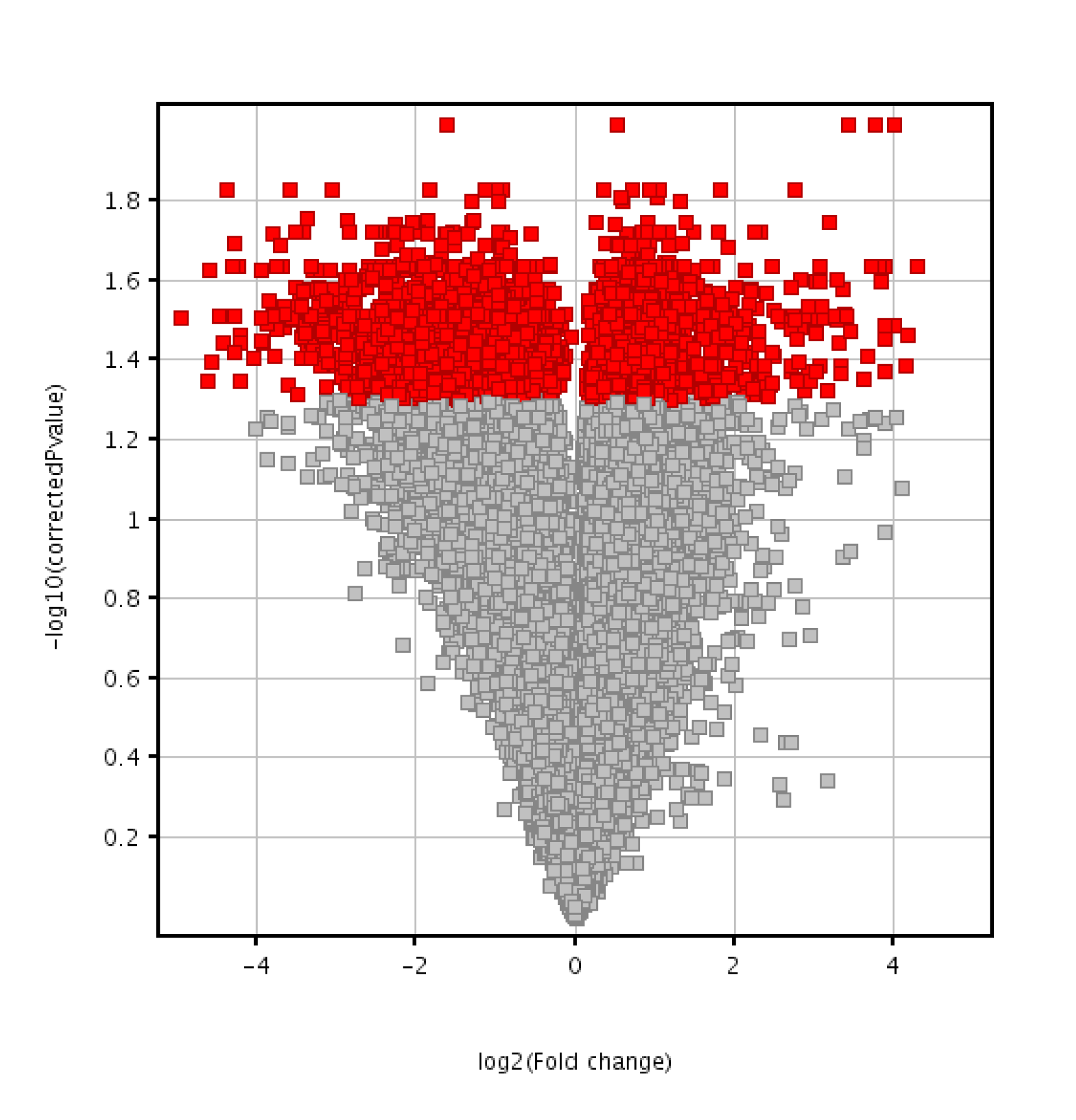

Supplement: Figure S6 — A volcano plot illustrated differentially regulated genes. (TIF) [file pone.0100542.s006.tif]
