# Supplementary material for: Cytotoxic and Pathogenic Properties of Klebsiella oxytoca Isolated from Laboratory Animals
Source: PLoS One. 2014 Jul 24;9(7):e100542. doi: 10.1371/journal.pone.0100542 (PMC4109914; doi:10.1371/journal.pone.0100542)
Supplement: Table S3 — Potential virulence genes found in K. oxytoca, 09-7231-1". (DOCX) [file pone.0100542.s009.docx]

**Supplementary Table 3**

| **Gene function** | **Locus ID** |
| --- | --- |
| **Type I secretion system** |  |
| - Type I secretion system ATPase | HMPREF9685_02044.1 |
| - HlyD family type I secretion membrane fusion   protein | HMPREF9685_02043.1 |
| - TolC family type I secretion outer membrane protein | [HMPREF9685_02045.1](http://www.broadinstitute.org/annotation/genome/Klebsiella_group/FeatureRedirect.html?sp=S7000007018469911) |
| - type 1 secretion C-terminal target domain (subclass) | [HMPREF9685_02046.1](http://www.broadinstitute.org/annotation/genome/Klebsiella_group/FeatureRedirect.html?sp=S7000007018469935) |
| **Type IV secretion system** |  |
| - Type IV pilus secretin PilQ | HMPREF9685_03905.1 |
| - R64 PilN family type IVB pilus formation outer membrane protein | HMPREF9685_05556.1 |
| - Type IV conjugative transfer system protein TraE | HMPREF9685_05446.1 |
| - Type IV conjugative transfer system protein | HMPREF9685_05452.1 |
| **Type VI secretion system** |  |
| - Type VI secretion lipoprotein | [HMPREF9685_01609.1](http://www.broadinstitute.org/annotation/genome/Klebsiella_group/FeatureRedirect.html?sp=S7000007018467244) |
| - Type VI secretion protein | [HMPREF9685_02966.1](http://www.broadinstitute.org/annotation/genome/Klebsiella_group/FeatureRedirect.html?sp=S7000007018478115) |
| - Type VI secretion protein | [HMPREF9685_01611.1](http://www.broadinstitute.org/annotation/genome/Klebsiella_group/FeatureRedirect.html?sp=S7000007018467238) |
| - Type VI secretion protein | [HMPREF9685_01627.1](http://www.broadinstitute.org/annotation/genome/Klebsiella_group/FeatureRedirect.html?sp=S7000007018467343) |
| - Type VI secretion protein | [HMPREF9685_01610.1](http://www.broadinstitute.org/annotation/genome/Klebsiella_group/FeatureRedirect.html?sp=S7000007018467255) |
| - Type VI secretion-associated protein | [HMPREF9685_02961.1](http://www.broadinstitute.org/annotation/genome/Klebsiella_group/FeatureRedirect.html?sp=S7000007018478081) |
| - EvpB/family type VI secretion protein | [HMPREF9685_02965.1](http://www.broadinstitute.org/annotation/genome/Klebsiella_group/FeatureRedirect.html?sp=S7000007018478108) |
| - Hcp1 family type VI secretion system effector | [HMPREF9685_02963.1](http://www.broadinstitute.org/annotation/genome/Klebsiella_group/FeatureRedirect.html?sp=S7000007018478094) |
| - ClpV1 family type VI secretion ATPase | [HMPREF9685_02962.1](http://www.broadinstitute.org/annotation/genome/Klebsiella_group/FeatureRedirect.html?sp=S7000007018478088) |
| - DotU family type IV/VI secretion system protein | [HMPREF9685_01626.1](http://www.broadinstitute.org/annotation/genome/Klebsiella_group/FeatureRedirect.html?sp=S7000007018467337) |
| - hypothetical protein | [HMPREF9685_01608.1](http://www.broadinstitute.org/annotation/genome/Klebsiella_group/FeatureRedirect.html?sp=S7000007018467250) |
| - rhs element Vgr protein | [HMPREF9685_01623.1](http://www.broadinstitute.org/annotation/genome/Klebsiella_group/FeatureRedirect.html?sp=S7000007018467328) |
| - rhs element Vgr protein | [HMPREF9685_00323.1](http://www.broadinstitute.org/annotation/genome/Klebsiella_group/FeatureRedirect.html?sp=S7000007018459508) |
| **Allantoin metabolism** |  |
| - HTH-type transcriptional activator AllS | HMPREF9685_02640.1 |
| - putative allantoin permease | [HMPREF9685_02632.1](http://www.broadinstitute.org/annotation/genome/Klebsiella_group/FeatureRedirect.html?sp=S7000007018474437) |
| - putative allantoin catabolism protein | [HMPREF9685_02628.1](http://www.broadinstitute.org/annotation/genome/Klebsiella_group/FeatureRedirect.html?sp=S7000007018474404) |
| - NCS1 nucleoside transporter | [HMPREF9685_02002.1](http://www.broadinstitute.org/annotation/genome/Klebsiella_group/FeatureRedirect.html?sp=S7000007018469604) |
| - HTH-type transcriptional repressor AllR | [HMPREF9685_02637.1](http://www.broadinstitute.org/annotation/genome/Klebsiella_group/FeatureRedirect.html?sp=S7000007018474473) |
| - allantoinase | HMPREF9685_02631.1 |
| **Fimbriae** |  |
| - Type 1 fimbriae regulatory protein fimB | HMPREF9685_03340.1 |
| - Type 1 fimbriae regulatory protein fimE | HMPREF9685_03341.1 |
| - MrkD | [HMPREF9685_03329.1](http://www.broadinstitute.org/annotation/genome/Klebsiella_group/FeatureRedirect.html?sp=S7000007018483601) |
| - FimH | [HMPREF9685_00535.1](http://www.broadinstitute.org/annotation/genome/Klebsiella_group/FeatureRedirect.html?sp=S7000007018460857) |
| - Fimbrial subunit type 3 | [HMPREF1024_02426.1](http://www.broadinstitute.org/annotation/genome/Klebsiella_group/FeatureRedirect.html?sp=S7000006942244379) |
| **Antibiotic/antimicrobial resistance** |  |
| - β-lactamase OXY-2 | [HMPREF9685_02585.1](http://www.broadinstitute.org/annotation/genome/Klebsiella_group/FeatureRedirect.html?sp=S7000007018474076) |
| - Penicillin-binding protein AmpH | [HMPREF9685_02832.1](http://www.broadinstitute.org/annotation/genome/Klebsiella_group/FeatureRedirect.html?sp=S7000007018476322) |
| - Penicillin-binding protein 2 | \| [**HMPREF9685_02401.1**](http://www.broadinstitute.org/annotation/genome/Klebsiella_group/FeatureRedirect.html?sp=S7000007018472652) \|  \| \| --- \| --- \| |
| - Drug resistance transporter, Bcr/CflA subfamily | [HMPREF9685_00743.1](http://www.broadinstitute.org/annotation/genome/Klebsiella_group/FeatureRedirect.html?sp=S7000007018462183) |
| - Drug:H+ antiporter-2 (14 Spanner) (DHA2) family drug resistance MFS transporter | [HMPREF9685_00624.1](http://www.broadinstitute.org/annotation/genome/Klebsiella_group/FeatureRedirect.html?sp=S7000007018461505) |
| - Putative multidrug resistance protein mdtD | [HMPREF9685_00445.1](http://www.broadinstitute.org/annotation/genome/Klebsiella_group/FeatureRedirect.html?sp=S7000007018460303) |
| - Quaternary ammonium compound-resistance protein sugE | [HMPREF9685_04682.1](http://www.broadinstitute.org/annotation/genome/Klebsiella_group/FeatureRedirect.html?sp=S7000007018510916) |
| - Multidrug resistance protein B | [HMPREF9685_03064.1](http://www.broadinstitute.org/annotation/genome/Klebsiella_group/FeatureRedirect.html?sp=S7000007018478885) |
| - Multidrug resistance protein MdtC | [HMPREF9685_00446.1](http://www.broadinstitute.org/annotation/genome/Klebsiella_group/FeatureRedirect.html?sp=S7000007018460317) |
| - Multidrug resistance protein MdtB | [HMPREF9685_04682.1](http://www.broadinstitute.org/annotation/genome/Klebsiella_group/FeatureRedirect.html?sp=S7000007018510916) |
| - Multidrug resistance protein mdtA | HMPREF9685_00448.1 |
| - Bicyclomycin resistance protein | [HMPREF9685_00337.1](http://www.broadinstitute.org/annotation/genome/Klebsiella_group/FeatureRedirect.html?sp=S7000007018459603) |
| - Macrolide-specific efflux protein macA | HMPREF9685_02130.1 |
| - Macrolide export ATP-binding/permease MacB | HMPREF9685_02129.1 |
| **Nitrate metabolism** |  |
| - Nitrate reductase | HMPREF9685_00796. |
| - Nitrate transporter NasD | [HMPREF9685_00794.1](http://www.broadinstitute.org/annotation/genome/Klebsiella_group/FeatureRedirect.html?sp=S7000007018462465) |
| - Respiratory nitrate reductase 1 gamma chain | [HMPREF9685_00804.1](http://www.broadinstitute.org/annotation/genome/Klebsiella_group/FeatureRedirect.html?sp=S7000007018462507) |
| - Respiratory nitrate reductase 2 gamma chain | [HMPREF9685_01405.1](http://www.broadinstitute.org/annotation/genome/Klebsiella_group/FeatureRedirect.html?sp=S7000007018466079) |
| - Nitrate reductase molybdenum cofactor assembly chaperone | [HMPREF9685_01404.1](http://www.broadinstitute.org/annotation/genome/Klebsiella_group/FeatureRedirect.html?sp=S7000007018466068) |
| - Nitrate reductase molybdenum cofactor assembly chaperone | HMPREF9685_01404.1 |
| - Nitrate reductase molybdenum cofactor assembly chaperone NarJ | [HMPREF9685_00803.1](http://www.broadinstitute.org/annotation/genome/Klebsiella_group/FeatureRedirect.html?sp=S7000007018462521) |
| - Nitrate/nitrite sensor protein narX | [HMPREF9685_00799.1](http://www.broadinstitute.org/annotation/genome/Klebsiella_group/FeatureRedirect.html?sp=S7000007018462498) |
| - Nitrate/nitrite response regulator protein narL | [HMPREF9685_00798.1](http://www.broadinstitute.org/annotation/genome/Klebsiella_group/FeatureRedirect.html?sp=S7000007018462491) |
| - Nitrite reductase [NAD(P)H] large subunit | [HMPREF9685_00795.1](http://www.broadinstitute.org/annotation/genome/Klebsiella_group/FeatureRedirect.html?sp=S7000007018462477) |
| - Molybdenum cofactor biosynthesis protein A | [HMPREF9685_02247.1](http://www.broadinstitute.org/annotation/genome/Klebsiella_group/FeatureRedirect.html?sp=S7000007018471440) |
| - Molybdenum cofactor biosynthesis protein B | [HMPREF9685_02246.1](http://www.broadinstitute.org/annotation/genome/Klebsiella_group/FeatureRedirect.html?sp=S7000007018471431) |
| - Molybdenum cofactor biosynthesis protein C | [HMPREF9685_02245.1](http://www.broadinstitute.org/annotation/genome/Klebsiella_group/FeatureRedirect.html?sp=S7000007018471424) |
| - Molybdenum transport system permease modB | [HMPREF9685_02280.1](http://www.broadinstitute.org/annotation/genome/Klebsiella_group/FeatureRedirect.html?sp=S7000007018471722) |
| - Molybdenum import ATP-binding protein ModC | [HMPREF9685_02279.1](http://www.broadinstitute.org/annotation/genome/Klebsiella_group/FeatureRedirect.html?sp=S7000007018471707) |
| **Iron metabolism** |  |
| - Iron-binding protein iscA | [HMPREF9685_00055.1](http://www.broadinstitute.org/annotation/genome/Klebsiella_group/FeatureRedirect.html?sp=S7000007018457274) |
| - Ferrous iron permease efeU | [HMPREF9685_01892.1](http://www.broadinstitute.org/annotation/genome/Klebsiella_group/FeatureRedirect.html?sp=S7000007018468873) |
| - Siderophore-iron reductase FhuF | [HMPREF9685_04978.1](http://www.broadinstitute.org/annotation/genome/Klebsiella_group/FeatureRedirect.html?sp=S7000007018513931) |
| - Ferrous iron transporter B | [HMPREF9685_04010.1](http://www.broadinstitute.org/annotation/genome/Klebsiella_group/FeatureRedirect.html?sp=S7000007018497042) |
| - Succinate dehydrogenase iron-sulfur subunit | [HMPREF9685_02332.1](http://www.broadinstitute.org/annotation/genome/Klebsiella_group/FeatureRedirect.html?sp=S7000007018472090) |
| - Ferrous iron transporter A | [HMPREF9685_04011.1](http://www.broadinstitute.org/annotation/genome/Klebsiella_group/FeatureRedirect.html?sp=S7000007018497068) |
| - Fumarate reductase iron-sulfur subunit | [HMPREF9685_04686.1](http://www.broadinstitute.org/annotation/genome/Klebsiella_group/FeatureRedirect.html?sp=S7000007018510986) |
| - Ferrous iron transporter C | [HMPREF9685_04009.1](http://www.broadinstitute.org/annotation/genome/Klebsiella_group/FeatureRedirect.html?sp=S7000007018497011) |
| - Iron-sulfur cluster insertion protein erpA | [HMPREF9685_05215.1](http://www.broadinstitute.org/annotation/genome/Klebsiella_group/FeatureRedirect.html?sp=S7000007018516169) |
| - Iron-sulfur cluster repair protein YtfE | [HMPREF9685_04733.1](http://www.broadinstitute.org/annotation/genome/Klebsiella_group/FeatureRedirect.html?sp=S7000007018511483) |
| - Iron(3+)-hydroxamate import system permease fhuB | [HMPREF9685_05211.1](http://www.broadinstitute.org/annotation/genome/Klebsiella_group/FeatureRedirect.html?sp=S7000007018516125) |
| - Iron(3+)-hydroxamate-binding protein fhuD | [HMPREF9685_05210.1](http://www.broadinstitute.org/annotation/genome/Klebsiella_group/FeatureRedirect.html?sp=S7000007018516116) |
| - Formate dehydrogenase-O iron-sulfur subunit | [HMPREF9685_04328.1](http://www.broadinstitute.org/annotation/genome/Klebsiella_group/FeatureRedirect.html?sp=S7000007018505780) |
| - Iron(3+)-hydroxamate import ATP-binding protein FhuC | [HMPREF9685_05209.1](http://www.broadinstitute.org/annotation/genome/Klebsiella_group/FeatureRedirect.html?sp=S7000007018516108) |
| - Formate dehydrogenase, nitrate-inducible, iron-sulfur subunit | [HMPREF9685_01386.1](http://www.broadinstitute.org/annotation/genome/Klebsiella_group/FeatureRedirect.html?sp=S7000007018465975) |
| **Citrate fermentation** |  |
| - Citrate synthase | [HMPREF9685_02336.1](http://www.broadinstitute.org/annotation/genome/Klebsiella_group/FeatureRedirect.html?sp=S7000007018472121) |
| - Citrate (Si)-synthase | [HMPREF9685_00459.1](http://www.broadinstitute.org/annotation/genome/Klebsiella_group/FeatureRedirect.html?sp=S7000007018460386) |
| - Citrate lyase alpha chain | [HMPREF9685_05069.1](http://www.broadinstitute.org/annotation/genome/Klebsiella_group/FeatureRedirect.html?sp=S7000007018514711) |
| - Citrate lyase subunit beta | [HMPREF9685_05068.1](http://www.broadinstitute.org/annotation/genome/Klebsiella_group/FeatureRedirect.html?sp=S7000007018514702) |
| - Citrate-sodium symporter | [HMPREF9685_05065.1](http://www.broadinstitute.org/annotation/genome/Klebsiella_group/FeatureRedirect.html?sp=S7000007018514678) |
| - Citrate-proton symporter | [HMPREF9685_02371.1](http://www.broadinstitute.org/annotation/genome/Klebsiella_group/FeatureRedirect.html?sp=S7000007018472377) |
| - Citrate lyase acyl carrier protein | [HMPREF9685_05067.1](http://www.broadinstitute.org/annotation/genome/Klebsiella_group/FeatureRedirect.html?sp=S7000007018514693) |
| - CCS family citrate carrier protein | [HMPREF9685_05088.1](http://www.broadinstitute.org/annotation/genome/Klebsiella_group/FeatureRedirect.html?sp=S7000007018514843) |
| - [Citrate (Pro-3S)-lyase] ligase | [HMPREF9685_05066.1](http://www.broadinstitute.org/annotation/genome/Klebsiella_group/FeatureRedirect.html?sp=S7000007018514685) |
| **Immune modulation** |  |
| - Ecotin | HMPREF9685_00321.1 |
| **Bacteria-bacteria competition** |  |
| \| - Colicin-A \|  \| \| --- \| --- \| | [HMPREF9685_02899.1](http://www.broadinstitute.org/annotation/genome/Klebsiella_group/FeatureRedirect.html?sp=S7000007018477298) |
| **Promotion of gastrointestinal tract colonization** |  |
| - Aerobic respiration control sensor protein ArcB | [HMPREF9685_03761.1](http://www.broadinstitute.org/annotation/genome/Klebsiella_group/FeatureRedirect.html?sp=S7000007018491852) |
| - Aerobic respiration control sensor protein ArcB | [HMPREF9685_05010.1](http://www.broadinstitute.org/annotation/genome/Klebsiella_group/FeatureRedirect.html?sp=S7000007018514177) |
| - Protein recA | [HMPREF9685_03075.1](http://www.broadinstitute.org/annotation/genome/Klebsiella_group/FeatureRedirect.html?sp=S7000007018478979) |
